# Amorphous Ni–Fe–Mo Suboxides Coupled with Ni Network as Porous Nanoplate Array on Nickel Foam: A Highly Efficient and Durable Bifunctional Electrode for Overall Water Splitting

**DOI:** 10.1002/advs.201902034

**Published:** 2020-02-05

**Authors:** Yong‐Ke Li, Geng Zhang, Wang‐Ting Lu, Fei‐Fei Cao

**Affiliations:** ^1^ Department of Chemistry College of Science Huazhong Agricultural University 430070 Wuhan P. R. China; ^2^ College of Resources and Environment Huazhong Agricultural University 430070 Wuhan P. R. China; ^3^ Institute for Interdisciplinary Research Jianghan University 430056 Wuhan P. R. China

**Keywords:** hierarchical porosity, hydrogen evolution reaction, oxide, oxygen evolution reaction, oxygen vacancy

## Abstract

It is a great challenge to fabricate electrode with simultaneous high activity for the hydrogen evolution reaction (HER) and the oxygen evolution reaction (OER). Herein, a high‐performance bifunctional electrode formed by vertically depositing a porous nanoplate array on the surface of nickel foam is provided, where the nanoplate is made up by the interconnection of trinary Ni–Fe–Mo suboxides and Ni nanoparticles. The amorphous Ni–Fe–Mo suboxide and its in situ transformed amorphous Ni–Fe–Mo (oxy)hydroxide acts as the main active species for HER and OER, respectively. The conductive network built by Ni nanoparticles provides rapid electron transfer to active sites. Moreover, the hydrophilic and aerophobic electrode surface together with the hierarchical pore structure facilitate mass transfer. The corresponding water electrolyzer demonstrates low cell voltage (1.50 V @ 10 mA cm^−2^ and 1.63 V @ 100 mA cm^−2^) with high durability at 500 mA cm^−2^ for at least 100 h in 1 m KOH.

## Graphical abstract

A high‐performance bifunctional electrode in the form of a porous nanoplate array vertically aligned on nickel foam for overall water splitting is provided. The porous nanoplate is built by the interconnection of trinary Ni–Fe–Mo suboxides and Ni nanoparticles, which is featured with amorphous active material and rapid electron/mass transfer.

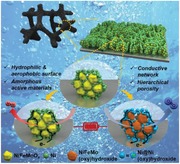

## 1 Introduction

The development of efficient electrocatalyst with earth‐abundant elements for hydrogen evolution reaction (HER) and oxygen evolution reaction (OER) is of great importance for the large‐scale production of hydrogen from water electrolysis.^1^ Although significant progress has been made,^2^ the achievement of high HER and OER activity on a single material is still a challenge, because the mechanism for HER is totally different from that of OER and vice versa, resulting in distinct requirements on the properties of electrocatalyst.^3^ Ni–Fe‐based^4^ and Ni–Mo‐based^5^ compounds have been proved to have high inherent activity for OER and HER, respectively, thus Ni–Fe–Mo‐based material is expected to be a promising candidate for overall water splitting. However, only limited work was focused on this field. The NiFeMo trimetallic film supported on nickel foam (NF) synthesized by Qin et al. showed good performance in overall water splitting;^6^ unfortunately, its smooth and compact surface hindered the further increment of activity due to the lack of active area. Wu et al. prepared NiFe foam supported MoS_2_/Fe_5_Ni_4_S_8_ heterostructures for water splitting;^7^ although superior OER activity was achieved, the HER activity was unsatisfactory, probably caused by the sluggish HER kinetics on MoS_2_.^8^ Generally, the catalytic efficiency of electrocatalyst is determined by its inherent activity and the number of active site. Introducing defects^9^ and reducing crystallinity^10^ have been proved to be effective strategies to increase the inherent activity of catalyst, both of which can lower the atomic coordination number and modify the electronic structure, making the adsorption and activation of reactant more easy. On the other hand, increasing the number of sites that are effective for reaction is of equal importance. In the electrolyzer, the electrocatalyst should be supported on a substrate (e.g., NF, carbon cloth (CC), ionic exchange membrane, etc.) to form an electrode, and HER/OER can only take place at the site contacting both electron and reactant. As a result, a high electronic conductivity and a high mass transfer efficiency are required for an advanced electrode. The former will facilitate the electron transfer between electrode substrate and active sites, while the latter will help the adsorption of reactants onto catalyst and prevent the blockage of active sites by bubbles generated.^11^

In this work, we designed a bifunctional electrode for overall water splitting, which is formed by depositing Ni–Fe–Mo‐based catalyst on the surface of NF. The catalyst presents in the form of porous nanoplate array, which is built by the interconnection of trinary amorphous Ni–Fe–Mo suboxides and Ni nanoparticles. The electrode is featured with amorphous active material and rapid electron/mass transfer properties, giving rise to high inherent activity and abundant active sites. The corresponding water electrolyzer exhibited low cell voltage and high durability in alkaline media.

## 2 Results and Discussion

**Scheme 1:**
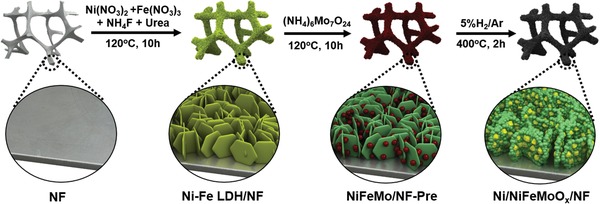
Illustration of fabricating process of Ni/NiFeMoO*_x_*/NF.

**Figure 1:**
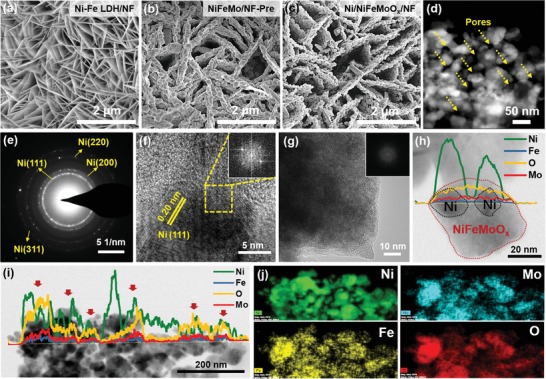
SEM images of a) Ni–Fe LDH/NF, b) NiFeMo/NF‐Pre, and c) Ni/NiFeMoO*_x_*/NF. d) HAADF‐STEM image and e) electron diffraction pattern of Ni/NiFeMoO*_x_*/NF. HRTEM images of f) Ni nanoparticle and g) NiFeMoO*_x_*. h–j) Elemental distribution of Ni/NiFeMoO*_x_*/NF. The inset in panels (f) and (g) display the fast Fourier transform (FFT) pattern of the corresponding image. Red arrows in panel (i) show locations of NiFeMoO*_x_* particles.

The electrode is prepared via a three‐step strategy (**Scheme** **1**). Ni–Fe layered double hydroxide (LDH) nanosheet array is first grown on NF with 3D open framework and high conductivity (**Figure** **1**a; Figures S1 and S2, Supporting Information).^12^ After the hydrothermal treatment in a solution containing (NH_4_)_6_Mo_7_O_24_, a solid molybdate containing Ni^2+^ cation and Fe^2+^ cation is deposited on the surface of Ni–Fe LDH (see Figure 1b and Figures S3–S15 in the Supporting Information for the characterization details), which is the precursor of the final product and thus is named NiFeMo/NF‐Pre. Finally, NiFeMo/NF‐Pre is reduced in 5%H_2_/Ar at 400 °C for 2 h to obtain Ni/NiFeMoO*_x_*/NF with atomic ratio of Mo, Ni, and Fe at 12.3:8.4:1 (Figure S16, Supporting Information). The scanning electron microscopy (SEM) and high‐angle annular dark field scanning TEM (HAADF‐STEM) images of Ni/NiFeMoO*_x_*/NF show that porous nanoplate array is deposited on the surface of NF (Figure 1c,d). The mercury intrusion analysis demonstrates the presence of megapores and macropores in electrode ranging from submillimeter to submicrometer (Figure S17a, Supporting Information), resulting from the open pores of NF and space among neighboring nanoplates in the array. The characteristic type‐IV N_2_‐sorption isotherms with distinct H_3_‐type hysteresis loops (Figure S17b, Supporting Information) further indicate the existence of large number of irregular mesopores in nanoplates.

**Figure 2:**
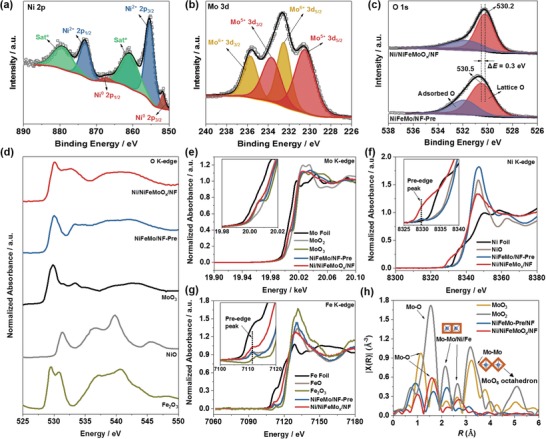
a–c) XPS spectra of Ni/NiFeMoO*_x_*/NF. XANES spectra at d) O K‐edge, e) Mo K‐edge, f) Ni K‐edge, and g) Fe K‐edge for NiFeMo/NF‐Pre, Ni/NiFeMoO*_x_*/NF, and referenced materials. h) FT‐EXAFS spectra at Mo K‐edge for NiFeMo/NF‐Pre, Ni/NiFeMoO*_x_*/NF, and MoO_3_.

Further analysis indicates that there are two components existing in the porous nanoplate: metallic Ni and Ni–Fe–Mo trinary oxides (NiFeMoO*_x_*). The X‐ray diffraction (XRD) (Figure S18, Supporting Information), selected area electron diffraction (SAED) (Figure 1e), high‐resolution TEM (HRTEM) (Figure 1f), and X‐ray photoelectron spectroscopy (XPS) (**Figure** **2**a) indicate the presence of metallic Ni in the reduced product. It is believed that metallic Ni is derived from Ni–Fe LDH, because Ni alloy can be obtained by reducing the powder of Ni–Fe LDH by H_2_ (Figure S18, Supporting Information). In addition to metallic Ni, another component exists in the nanoplate. The elemental line profiles show that Mo, Fe, and O element exist in the second component, and Ni element is overlapped with them where no Ni particles contacted (Figure 1h; Figure S19, Supporting Information), suggesting Ni element is also present in this component. The line shape of the O K‐edge X‐ray absorption near edge structure (XANES) of Ni/NiFeMoO*_x_*/NF is like that of MoO_3_, but quite different from that of NiO and Fe_2_O_3_ (Figure 2d), indicating that the second component should be an oxide with a structure similar with MoO_3_.^13^ The Mo(0) signal is not detected by XPS (Figure 2b), and the adsorption edge in the Mo K‐edge XANES of Ni/NiFeMoO*_x_*/NF locates between MoO_2_ and MoO_3_ (Figure 2e), proving Mo exists in an ionic state with average valence between +6 and +4. The Ni XPS (Figure 2a) together with the intense white line in the Ni K‐edge XANES (just like that of NiO) indicates the existence of Ni^2+^ species (Figure 2f).^14^ As for Fe, the similar intensity of white line with FeO and the same location of pre‐edge peak with FeO (7112 eV) (Figure 2g),^15^ suggesting that large number of Fe^2+^ ions reside in Ni/NiFeMoO*_x_*/NF. As a result, the second component in nanoplate should be a Ni–Fe–Mo trinary oxide (NiFeMoO*_x_*) with a basic structure like MoO_3_, where Ni^2+^ and Fe^2+^ ions locate at the site similar with that of Mo in MoO_3_. Based on the above results, it can be concluded that Ni–Fe LDH and Ni–Fe–molybdate in NiFeMo/NF‐Pre is transformed to Ni and NiFeMoO*_x_*, respectively, during H_2_ reduction.

Furthermore, it is found that NiFeMoO*_x_* possesses an amorphous structure and abundant oxygen vacancies. As shown in Figure 1e and Figure S18 in the Supporting Information, only Ni diffraction patterns are found in the patterns of XRD and SAED; and only lattice fringes ascribed to Ni are detected by HRTEM (Figure 1f,g), all of which prove the amorphous nature of NiFeMoO*_x_*. The amorphous structure is usually associated with defects. The presence of Mo^5+^ species in NiFeMoO*_x_* (Figure 2b) implies that the content of oxygen will not conform to stoichiometric ratio. Moreover, a negative shift of 0.3 eV on binding energy of lattice‐O in comparison with NiFeMo/NF‐Pre (Figure 2c) suggests the presence of O vacancies in Ni/NiFeMoO*_x_*/NF.^16^ The Raman peaks at 887 and 892 cm^−1^ can be ascribed to the Mo–O stretching mode in MoO*_x_* with oxygen deficiency (Figure S8, Supporting Information).^17^ It is well known that the pre‐edge peak in the Ni or Fe K‐edge XANES is assignable to an inner‐atomic 1s–3d electronic transition and is determined by dipole selection rules.^18^ The weak intensity of Ni and Fe pre‐edge peaks for NiFeMo/NF‐Pre (Figure 2f,g) manifests that Ni and Fe are in fact existing in symmetric NiO_6_ and FeO_6_ octahedral units in which the electronic transition from 1s to 3d is dipole‐forbidden.[qv: 18a] However, both Ni and Fe pre‐edge peaks are obviously enhanced after H_2_ reduction (Figure 2f,g), which can be ascribed to the decrease of symmetry of Ni/Fe–O coordination system induced by oxygen vacancies in Ni/NiFeMoO*_x_*/NF.[qv: 18b,19] In the spectra of Fourier transformed extended X‐ray adsorption fine structure (FT‐EXAFS) of Mo (Figure 2h), phase‐uncorrected peaks at ≈1.0 Å (Mo–O1) and ≈1.6 Å (Mo–O2) of NiFeMo/NF‐Pre are ascribed to the Mo—O bond with various atom distance in the MoO_6_ unit;^20^ the doublet peaks at ≈2.1 and ≈2.6 Å can be assigned to the Mo–Mo coordination of two neighboring edge‐shared MoO_6_ units and/or Mo–Ni and/or Mo–Fe coordination.[qv: 20a,21] In MoO_2_ and MoO_3_, the peak at ≈3.2 Å comes from the Mo–Mo coordination of two neighboring corner‐shared MoO_6_ units.^20^, ^21^ The H_2_ reduction makes the Mo–O2 peak more intense than that of Mo–O1 peak, and moves the Mo—O2 bond distance to the negative direction with respect to NiFeMo‐Pre/NF and MoO_3_ (Figure S20, Supporting Information). Such changes of EXAFS can be attributed to the formation of oxygen deficiency in the material by H_2_ reduction.[qv: 20a,22] Additionally, the absence of Mo–Mo scattering at distance longer than ≈3.0 Å further manifests the amorphous nature of NiFeMoO*_x_*.^21^ The above results confirm the presence of amorphous NiFeMoO*_x_* with oxygen vacancies in Ni/NiFeMoO*_x_*/NF. Furthermore, Figures 1i,j and Figure S21 in the Supporting Information demonstrate that Ni particles forms a network and interconnects with NiFeMoO*_x_* to compose a porous nanoplate. This novel architecture will facilitate the electron transfer from NF to catalytic active sites with the help of highly conductive Ni network, and the presence of pores will increase the number of active sites exposed.

**Figure 3:**
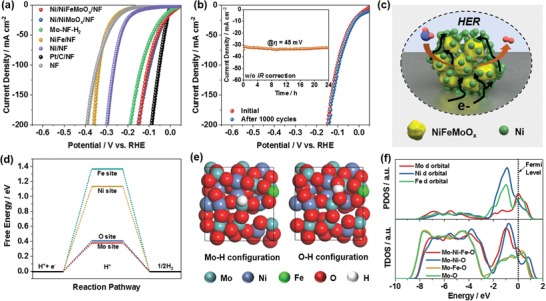
a) *iR*‐corrected polarization curves of various electrodes for HER in 1 m KOH. b) *iR*‐corrected polarization curves of Ni/NiFeMoO*_x_*/NF before and after 1000 potential cycles between 0.067 and −0.174 V (*iR* uncorrected); (inset) the *i*–*t* curve of Ni/NiFeMoO*_x_*/NF at η = 45 mV (*iR* uncorrected) for 24 h. c) Schematic illustration of Ni/NiFeMoO*_x_*/NF during HER. d) The calculated hydrogen adsorption free energy on different sites in NiFeMoO*_x_*. e) The calculated adsorption configuration of H* on Mo and O sites. f) The calculated partial DOS (PDOS) of Mo, Ni, and Fe in NiFeMoO*_x_* and total DOS (TDOS) of various material systems.

The H_2_ reduction not only changes the structure but also transforms the electrode to be highly electrochemical active in term of double layer capacitance (*C*_dl_) and overpotential (η) for HER (Figure S22, Supporting Information). Specifically, Ni/NiFeMoO*_x_*/NF achieves the hydrogen evolving current of 10 and 100 mA cm^−2^ at overpotential of 22 and 117 mV in 1 m KOH (**Figure** **3**a), respectively, with a Tafel slope at 76 mV dec^−1^ (Figure S22, Supporting Information), which outperforms numerous HER electrodes reported recently (Table S1, Supporting Information). In order to evaluate the intrinsic activity, the hydrogen evolving current (*j*) of Ni/NiFeMoO*_x_*/NF was normalized by its *C*_dl_, and the *j*/*C*_dl_ obtained should be positively correlated with the area‐specific activity. The *j*/*C*_dl_ of Ni/NiFeMoO*_x_*/NF for HER at overpotential of 50 and 100 mV is 0.061 and 0.17 mA mF^−1^, respectively, which is superior or comparable to that of MoS_2_–Ni_3_S_2_/NF (≈0.021 mA mF^−1^ at η = 50 mV),^23^ Cu@NiFe LDH/CF (≈0.084 mA mF^−1^ at η = 100 mV),^24^ Ni_3_V‐VN/NF (≈0.082 mA mF^−1^ at η = 100 mV),^25^ MoNi_4_‐450/NF (≈0.094 mA mF^−1^ at η = 50 mV),[qv: 5f] and porous MoO_2_/NF (≈0.064 mA mF^−1^ at η = 50 mV).^26^ In addition to superior activity, Ni/NiFeMoO*_x_*/NF demonstrates a high stability under both constant and dynamic potential conditions (Figure 3b; Figure S23a, Supporting Information) with morphology and composition well maintained (Figures S24–S26, Supporting Information).

We found that Mo^5+^ and/or O vacancy played an important role in the catalysis of HER. No matter the Mo‐free Ni/NF (Figure S27, Supporting Information) and NiFe/NF (Figure S28, Supporting Information) respectively reduced from Ni(OH)_2_/NF and Ni–Fe LDH/NF, or the Ni–Fe–Mo electrode with lower Mo content (Figure S29, Supporting Information), or the electrode with most Mo^5+^ and O vacancies removed by continuous oxygen evolution (Figure S30, Supporting Information), they all show degraded HER activity. Additionally, both electrodes obtained at 300 and 500 °C with lower content of Mo^5+^ and O vacancies exhibit poorer activity for HER (Figures S31–S33, Supporting Information). In alkaline media, the HER process is initialized by the dissociation of H_2_O on the surface of catalyst to form adsorbed H atom (H*), which is followed by the electrochemical desorption or recombination of H* to generate H_2_ molecule.[qv: 2b] The O vacancy in the material has been suggested to have strong ability in the dissociation of H_2_O to *OH and H*,[qv: 16a,27] and it is widely accepted that the optimal catalytic site for H_2_ formation should have an adsorption free energy of H* (Δ*G*_H*_) close to 0 eV, i.e., the H* is adsorbed neither too strongly nor too weakly.^28^ In order to distinguish the most active site for HER in NiFeMoO*_x_*, Δ*G*_H*_ on different sites is evaluated by density functional theory (DFT) calculation. According to structure characterizations, the NiFeMoO*_x_* system is built based on MoO_3_, and part of the Mo atoms are replaced by Ni or Fe, and part of the O atoms are removed to create O vacancies (Figure S34 Supporting Information). For the sake of simulating the amorphous nature of NiFeMoO*_x_*, the model is relaxed at 1000 K, so the ordered atomic arrangement is damaged. As shown in Figure 3d, the Δ*G*_H*_ on Mo, O, Ni, and Fe site is 0.39, 0.40, 1.14, and 1.37 eV, respectively, indicating that the interaction between Ni/Fe and H* is too weak, and thus Mo and O sites are more active (Figure 3e), which can be supported by the higher density of state (DOS) on Mo atom with respect to Ni and Fe at the Fermi level (Figure 3f). The introduction of Ni and Fe into the Mo–O system may improve the electronic conductivity of the material because the total DOS of the system at the Fermi level is enhanced (Figure 3f). Moreover, it is found that the interaction between Mo and H* is too strong in the absence of Ni and Fe (Δ*G*_H*_ = −1.23 eV), but the incorporation of Ni/Fe can weaken this interaction especially for Ni (Figures S35 and S36, Supporting Information): the Δ*G*_H*_ on Mo in the Fe‐free Mo–Ni–O system is −0.51 eV, which is much lower than that in the Ni‐free Mo–Fe–O system (−0.86 eV). This may explain the fact that the Ni/NiMoO*_x_*/NF electrode fabricated in the absence of Fe presents similar HER performance with that of Ni/NiFeMoO*_x_*/NF (Figure 3a; Figure S37, Supporting Information). Additionally, the DFT calculation reveals that the synergistic effect between NiFeMoO*_x_* and metallic Ni is not significant (Figure S38, Supporting Information), further indicating NiFeMoO*_x_* is the main active material for HER. Furthermore, the morphology of active materials is found to affect the performance of HER. The electrochemical specific surface area (ECSA) of Ni/NiFeMoO*_x_*/NF (652 m^2^ g^−1^) is similar with that of Ni/NiMoO*_x_*/NF (629 m^2^ g^−1^), but larger than 498 m^2^ g^−1^ of the electrode without nanoplate array architecture (NF–Mo–H_2_) (Figures S39 and S40, Supporting Information), which may result in the lower HER activity of NF–Mo–H_2_ (Figure 3a).

**Figure 4:**
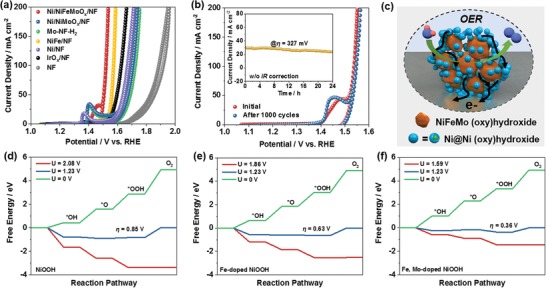
a) *iR*‐corrected CV curves of various electrodes for OER in 1 m KOH. b) *iR*‐corrected CV curves of Ni/NiFeMoO*_x_*/NF before and after 1000 potential cycles between 1.067 and 1.7 V (*iR* uncorrected); (inset) the *i*–*t* curve of Ni/NiFeMoO*_x_*/NF at η = 327 mV (*iR* uncorrected) for 24 h. c) Schematic illustration of Ni/NiFeMoO*_x_*/NF during OER. Free energy diagrams of OER on d) Ni, e) Ni–Fe, and f) Ni–Fe–Mo system.

The catalytic performance of Ni/NiFeMoO*_x_*/NF for OER was also evaluated (**Figure** **4**a). The overpotential of Ni/NiFeMoO*_x_*/NF at 10 and 100 mA cm^−2^ is 255 and 289 mV in 1 m KOH, respectively, with a Tafel slope as low as 35 mV dec^−1^ (Figure S41, Supporting Information). This performance is superior or comparable to many noble‐metal free OER electrodes (Table S2, Supporting Information). Different to the significance of Mo species in HER, Fe plays an essential role in the OER process. The Fe‐free Ni/NF, Ni/NiMoO*_x_*/NF, and Mo–NF–H_2_ electrode show much lower OER activity, whereas NiFe/NF shows performance approaching Ni/NiFeMoO*_x_*/NF (Figure 4a; Figure S41, Supporting Information). Moreover, the reduction temperature (300–500 °C) has little effect on the OER activity of Ni–Fe–Mo electrode (Figure S33b, Supporting Information). All these results evidence the importance of Fe to the OER activity of electrode.

The Ni/NiFeMoO*_x_*/NF electrode also presents stable performance during OER process (Figure 4b; Figure S23b, Supporting Information). However, significant structure transformation is observed after long‐term O_2_ evolution, indicating that the actual active species for OER would not be the same with that of HER. The Mo content is remarkably reduced from 73% to 1.7% only with Mo^6+^ species left, and O vacancies in the material are removed (Figure S42, Supporting Information); meanwhile, all Mo–O Raman peaks disappear (Figure S43, Supporting Information). The elemental analysis to the electrolyte confirms the obvious dissolution of Mo from electrode (Figure S44, Supporting Information). It is believed that the high electrode potential during OER can oxidize low‐valent Mo species to soluble Mo^6+^ compounds. The remaining components of NiFeMoO*_x_* are reconstructed to form a structure with uniform distribution of Ni, Fe, and Mo (Figure S45, Supporting Information). The O K‐edge XANES spectrum of the electrode after oxygen evolution shows a line shape quite similar to Ni–Fe LDH/NF (Figure S46, Supporting Information), and the Raman spectrum presents a distinct peak at ≈550 cm^−1^ (Figure S43, Supporting Information), which can be ascribed to Ni‐based (oxy)hydroxide.^4^ It has been reported that metal oxides usually transformed to (oxy)hydroxide during OER owing to the high electrode potential and oxidizing environment,^29^ and the positive shift of Mo and Fe K‐edge XANES spectra confirms the valence increase of elements in the electrode (Figure S46, Supporting Information). Therefore, it can be concluded that NiFeMoO*_x_* is in situ transformed into NiFeMo (oxy)hydroxide after oxygen evolution process. Moreover, the electron diffraction and HRTEM reveal that the NiFeMo (oxy)hydroxide has an amorphous structure (Figure S47, Supporting Information). As for the Ni nanoparticles in Ni/NiFeMoO*_x_*/NF, the Ni diffraction rings and lattice fringe are still observed (Figure S47, Supporting Information), whereas the Ni(0) XPS signal disappeared (Figure S42, Supporting Information), indicating that the surface of Ni nanoparticles is also oxidized to form an amorphous Ni (oxy)hydroxide, which is supported by TEM (Figure S47, Supporting Information). Considering the fact that Fe could significantly enhance the OER activity of Ni‐based materials, it is believed that the in situ formed amorphous NiFeMo (oxy)hydroxide is the main active species for OER (Figure 4c).

The effect of Fe and Mo on the OER activity in the electrode was analyzed by DFT+U method. Due to the high electrode potential during OER, NiOOH (i.e., a high‐valence Ni species), Fe‐doped NiOOH, and Fe, Mo‐codoped NiOOH was employed to model the surface of Ni, Ni–Fe, Ni–Fe–Mo system during OER, respectively (Figure S48, Supporting Information), and the conventional four‐step mechanism (*—OH, *—O, *—OOH, and *+O_2_; * denotes the active site) was adopted to describe the OER process.[qv: 10b] For the system without Fe and Mo, the process from *OOH to O_2_ has the highest free energy difference (Figure 4d), which can be regarded as the rate determining step of OER. The overpotential calculated on NiOOH is 0.85 V. When Fe was incorporated into NiOOH, the rate determining step is not changed, but the overpotential was decreased to 0.63 V (Figure 4e), indicating the improved OER activity of Ni–Fe system. In the Ni–Fe–Mo system, the overpotential is further decreased to 0.36 V (Figure 4f), which is consistent with the result that the activity of Ni/NiFeMoO*_x_*/NF is higher than that of NiFe/NF (Figure 4a).

The electrochemical activity of Ni/NiFeMoO*_x_*/NF is believed to be benefited from its unique architecture. The electronic conductivity of catalyst is very important to the accomplishment of catalytic process of electrochemical reactions. In order to increase the electron transfer ability of catalyst, the construction of electron pathway is an effective approach. For example, Cu nanowire,^24^ carbon sheath,^30^ and Ni_3_S_2_ nanosheet^31^ have been used as conductive media to help the transfer of electron between substrate and catalytic active species. Here, in this work, we also built a high‐speed electron conductive route by using conductive Ni network in the electrode, which facilitates the electron transfer between NF and active site in NiFeMoO*_x_* or NiFeMo (oxy)hydroxide (Figures 3c and 4c). Moreover, the hierarchical pore structure (Figure S17, Supporting Information) helps to increase mass transfer rate and expose active sties. In addition, the electrode shows a hydrophilic and aerophobic surface simultaneously (Figure S49, Supporting Information), which enhances the interaction between electrode and reactant in water, and accelerates the release of H_2_/O_2_ from electrode, avoiding the blockage of active sites by bubbles generated.[qv: 11a] It is found that the mass loading of active material on Ni/NiFeMoO*_x_*/NF is comparable to many electrodes reported, but Ni/NiFeMoO*_x_*/NF has much larger *C*_dl_ (Tables S1 and S2, Supporting Information), indicating a high active surface area on Ni/NiFeMoO*_x_*/NF, which probably comes from the unique architecture of Ni/NiFeMoO*_x_*/NF. Furthermore, Figure S50 in the Supporting Information shows that the electrode overpotential at high current densities for Ni/NiFeMoO*_x_*/NF is not raised obviously with the increase of scan rate up to 500 mV s^−1^, proving its superior charge and mass transport properties. Importantly, the array morphology and hydrophilic/aerophobic surface can be maintained after durability testing for both HER and OER (Figure S51, Supporting Information).

**Figure 5:**
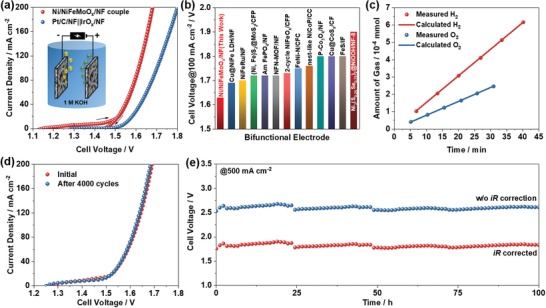
a) *iR*‐corrected polarization curves of Ni/NiFeMoO*_x_*/NF and Pt/C||IrO*_x_* electrolyzer in 1 m KOH. b) Comparison of the cell voltage of Ni/NiFeMoO*_x_*/NF with other bifunctional electrodes: Cu@NiFe LDH/NF,^24^ NiFeRu LDH/NF,^34^ (Ni, Fe)S_2_@MoS_2_/CFP,^35^ Am FePO_4_/NF,[qv: 10b] NFN‐MOF/NF,^36^ two‐cycle NiFeO*_x_*/CFP,^37^ FeNi‐N/CFC,^38^ nest‐like NiCoP/CC,^39^ P‐Co_3_O_4_/NF,^40^ CF‐Cu@CoS*_x_*/CF,[qv: 10e] FeS/IF,^41^ and Ni_3_(S_0.25_Se_0.75_)_2_@NiOOH/NF‐8.^42^ c) Comparison between the amount of H_2_/O_2_ collected and calculated for Ni/NiFeMoO*_x_*/NF. d) Polarization curves of Ni/NiFeMoO*_x_*/NF electrolyzer before and after 4000 potential cycles between 1.2 and 1.887 V (*iR* uncorrected). e) Chronopotentiometric response of Ni/NiFeMoO*_x_*/NF electrolyzer at 500 mA cm^−2^.

A water electrolyzer was constructed by two pieces of Ni/NiFeMoO*_x_*/NF electrode to evaluate its overall water splitting performance. The cell voltage of Ni/NiFeMoO*_x_*/NF couple required to achieve the current density of 10 and 100 mA cm^−2^ is 1.50 and 1.63 V in 1 m KOH (**Figure** **5**a), respectively, which is superior to that of benchmark Pt/C||IrO*_x_* couple. The high activity for both HER and OER enables Ni/NiFeMoO*_x_*/NF to be one of the most active bifunctional electrodes for overall water splitting in alkaline media (Figure 5b; Table S3, Supporting Information). The faradaic efficiency for both HER and OER on Ni/NiFeMoO*_x_*/NF is very close to 100%, indicating a high selectivity toward water splitting (Figure 5c). Furthermore, the Ni/NiFeMoO*_x_*/NF‐based electrolyzer presents excellent durability under both transient and steady conditions. The polarization curves of Ni/NiFeMoO*_x_*/NF couple are almost overlapped before and after 4000 potential cycles (Figure 5d). At 500 mA cm^−2^, a current density even larger than that of the practical alkaline water electrolyzer (100–300 mA cm^−2^),^32^ the electrolyzer performs steadily for at least 100 h (Figure 5e), which is also better than many other bifunctional electrodes (Table S3, Supporting Information). As a demonstration, the Ni/NiFeMoO*_x_*/NF electrolyzer works well powered by a commercial battery (≈1.5 V) or a solar cell in the open air (Figure S52 and Videos S1 and S2, Supporting Information), indicating a potential application of hydrogen producing in the future.

## 3 Conclusions

In this work, a highly efficient and durable bifunctional Ni/NiFeMoO*_x_*/NF electrode for overall water splitting in alkaline media is provided, and its superior performance can be attributed to the following points: 1) amorphous NiFeMoO*_x_* with oxygen vacancies and its in situ transformed amorphous NiFeMo (oxy)hydroxide have high inherent activity and abundant catalytic sites for HER and OER, respectively; 2) the metallic Ni network provides rapid electron transfer between NF and active sites; 3) the hierarchical pore structure and a hydrophilic and aerophobic surface elevate mass transfer; 4) a porous and binder‐free electrode structure prevents the peeling off of electrocatalyst from substrate during gas production. This work not only provides an advanced electrode for HER/OER, but also gives rise to a promising electrode/catalyst design strategy for water electrolyzer with potential application in the future.

## 4 Experimental Section

##### Reagents

Nickel foam (1.0 mm in thickness) and carbon cloth (W0S1002) were purchased from Li Zhi Yuan (Taiyuan, China) and CeTech (Taiwan, China), respectively. Ammonium heptamolybdate tetrahydrate ((NH_4_)_6_Mo_7_O_24_·4H_2_O, AHM) was provided by Strem Chemicals. All the other chemicals used in the synthesis came from Sinopharm Chemical Reagent Co., Ltd. with analytical grade. All reagents were used as received without further treatment. Ultrapure water (18.25 MΩ cm) was used in all experiments.

##### Preparation of Ni–Fe LDH/NF and Ni(OH)_2_/NF Electrode

NF was pretreated before use by sonication in ethanol and then 10% HCl aqueous solution followed by washing thoroughly with water and drying. Ni–Fe LDH/NF was prepared as follows:^12^ Ni(NO_3_)_2_·6H_2_O (2.25 mmol), Fe(NO_3_)_3_·9H_2_O (0.225 mmol), NH_4_F (4 mmol), and urea (10 mmol) were dissolved into water (40 mL), which was transferred into a Teflon‐lined stainless‐steel autoclave (50 mL), and then a piece of pretreated NF (2.5 cm × 6 cm) was added. After maintaining at 120 °C for 10 h, the autoclave was allowed to cool down naturally, and Ni–Fe LDH/NF electrode was finally obtained after washing and drying. The mass loading of Ni–Fe LDH on NF was 2.1 mg cm^−2^. When Fe salt was removed in the synthesis and the amount of Ni(NO_3_)_2_·6H_2_O was increased to 2.5 mmol, Ni(OH)_2_/NF electrode was obtained.

##### Preparation of NiFeMo/NF‐Pre Electrode

An aqueous solution was prepared by dissolving AHM (8.9 mmol) into water (40 mL), which was then transferred into an autoclave (50 mL) followed by the introduction of Ni–Fe LDH/NF electrode. The autoclave was maintained at 120 °C for several hours, and then cooled down. The product was washed thoroughly by water, and then dried to obtain NiFeMo/NF‐Pre. Except where otherwise stated, the NiFeMo/NF‐Pre electrode was the one prepared with 10 h duration, on which the mass loading of the deposit was 4.0 mg cm^−2^.

##### Preparation of Ni/NiFeMoO*_x_*/NF Electrode

The NiFeMo/NF‐Pre electrode was treated in a tubular oven under H_2_/Ar (v/v, 5/95) at 300, 400, or 500 °C for 2 h. After the oven was cooled down naturally, Ni/NiFeMoO*_x_*/NF electrode was obtained. Except where otherwise stated, the Ni/NiFeMoO*_x_*/NF electrode was the one prepared at 400 °C, and the mass loading of the active material on NF was 1.8 mg cm^−2^.

##### Preparation of NiMo/NF‐Pre and Ni/NiMoO*_x_*/NF Electrode

The NiMo/NF‐Pre electrode was prepared by treating Ni(OH)_2_/NF with AHM via similar procedures with that of NiFeMo/NF‐Pre. The NiMo/NF‐Pre electrode was treated in a tubular oven under H_2_/Ar (v/v, 5/95) at 400 °C for 2 h. After the oven was cooled down naturally, Ni/NiMoO*_x_*/NF electrode was obtained, and the mass loading of the active material on NF was 1.8 mg cm^−2^.

##### Preparation of NF–Mo and NF–Mo–H_2_ Electrode

The NF–Mo electrode was prepared by similar procedures with that of NiFeMo/NF‐Pre except that NF instead of Ni–Fe LDH/NF was used in the synthesis. The NF–Mo electrode was treated in a tubular oven under H_2_/Ar (v/v, 5/95) at 400 °C for 2 h. After the oven was cooled down naturally, NF–Mo–H_2_ electrode was obtained, and the mass loading of the active material on NF was 1.5 mg cm^−2^.

##### Preparation of NiFe/NF and Ni/NF Electrode

The Ni–Fe LDH/NF or Ni(OH)_2_/NF electrode was treated in a tubular oven under H_2_/Ar (v/v, 5/95) at 400 °C for 2 h. After the oven was cooled down naturally, NiFe/NF or Ni/NF electrode was obtained.

##### Preparation of Ni–Fe LDH/CC and Ni–Fe LDH–Mo/CC Electrode

The Ni–Fe LDH/CC electrode was prepared by similar procedures with that of Ni–Fe LDH/NF except that CC instead of NF was used in the synthesis. The Ni–Fe LDH–Mo/CC electrode was prepared followed by similar procedures with that of NiFeMo/NF‐Pre except that Ni–Fe LDH/CC instead of Ni–Fe LDH/NF was used in the synthesis.

##### Physical Characterizations

SEM images were taken by a Hitachi SU8010 microscope and the composition of the material deposited on the surface of NF was tested by peeling off the deposit from NF by sonication and then measured by the EDS detector equipped on SU8010. The HRTEM analysis was performed on a JEOL JEM‐2100F microscope. The scanning TEM (STEM) image and elemental mapping/line profile were obtained on an aberration‐corrected FEI Titan Themis 200 microscope equipped with Bruker Super‐X energy spectrometer. XRD patterns were recorded on a Bruker D8 Advance X‐ray diffractometer with Cu Kα radiation. For XPS testing, the as‐prepared or used electrodes were washed thoroughly by water and dried at 60 °C overnight under vacuum, and then XPS spectra were recorded on a Thermo Scientific ESCALAB 250Xi spectrometer using Mg Kα radiation, and the high‐resolution spectrum of an element was collected at a pass energy of 30 eV with a step size of 0.05 eV. The synchrotron‐based XANES at the O K‐edge and Mo/Ni/Fe K‐edge was measured at the beamline BL08U and BL14W1 station of Shanghai Synchrotron Radiation Facility, respectively. The EXAFS at the Mo K‐edge was measured at the beamline 1W1B station of the Beijing Synchrotron Radiation Facility. The mercury intrusion analysis and N_2_‐sorption analysis were carried out on Micromeritics AutoPore IV and ASAP 2460 analyzer, respectively. The pore size distribution from N_2_‐sorption was provided based on the Barrett–Joyner–Halenda (BJH) model. The inductively coupled plasma‐mass spectrometry (ICP‐MS) was performed on an Agilent 7900 spectrometer. Raman spectra were obtained on a Renishaw inVia spectrometer with exciting laser at 633 nm. Contact angles of electrode were measured on a Dataphysics OCA15EC contact angle meter.

##### Electrochemical Analysis

The electrochemical analysis was conducted on a CHI‐760E electrochemical station with a three‐electrode system at room temperature. Graphite rod and saturated calomel electrode (SCE) were used as the counter electrode and reference electrode, respectively. The electrode potential was presented versus the reversible hydrogen electrode (RHE) unless otherwise noted. The electrolyte used was 1 m KOH. The activity of electrode toward HER and OER was evaluated by linear sweep voltammetry (LSV) and cyclic voltammetry (CV) at a scan rate of 5 mV s^−1^, respectively. The ohmic resistance of the electrochemical system was measured by electrochemical impedance spectrum (EIS). The electrode potential was corrected by *iR* drop: |*E*_true_| = |*E*_measure_| − |*i*_measure_| × *R*, where *i*_measure_ and *E*_measure_ are the measured current and electrode potential, respectively; *R* is the ohmic resistance measured by EIS. The *C*_dl_ of electrode was calculated on the basis of CV curves recorded at various scan rates (1–10 mV s^−1^) in a non‐Faradaic region. The ECSA of active material on electrode was calculated based on *C*_dl_ and mass loading (*L*): ECSA = *C*_dl_/40 µF cm^−2^/*L*, where 40 µF cm^−2^ was assumed to be the capacitance of a flat surface.^33^ The working electrode of 20%Pt/C or IrO*_x_* (Johnson Matthey) was prepared by ultrasonicating catalyst powder in a mixture of ethanol and Nafion ionomer for ≈30 min followed by dipping the slurry onto a piece of NF. The two‐electrode water electrolyzer was performed in 1 m KOH at room temperature. The polarization curve of the electrolyzer was recorded by CHI‐760E at a scan rate of 5 mV s^−1^. The cell voltage was also given after *iR* correction. The faradaic efficiency for HER and OER on the electrode was measured based on the amount of gas collected by displacement of water and that calculated from electric quantity during water splitting.

##### DFT Calculation

For the HER process, the NiFeMoO*_x_* system was built based on MoO_3_, and part of the Mo atoms were replaced by Ni or Fe, and part of the O atoms were removed to create O vacancies. Thus, the atomic ratio of Ni:Fe:Mo:O in the model of NiFeMoO*_x_* was 8:1:12:42 (see the Supporting Information for the calculation detail). In order to simulate the amorphous nature of NiFeMoO*_x_*, the model was relaxed at 1000 K, so the ordered atomic arrangement was damaged. Then, the surface of amorphous NiFeMoO*_x_* was cut along the (101) direction, and the vacuum space along the *z*‐direction was set to be 15 Å, which was enough to avoid interaction between the two neighboring images. Each atom in the storage models was allowed to relax to the minimum in the enthalpy without any constraints. Then, H atom was absorbed on the substrate surface in different sites. The first principles calculations in the framework of DFT, including structural and electronic performances, were carried out based on the Cambridge Sequential Total Energy Package known as CASTEP. The exchange–correlation functional under the generalized gradient approximation (GGA) with norm‐conserving pseudopotentials and Perdew–Burke–Ernzerhof functional was adopted to describe the electron–electron interaction. An energy cutoff of 750 eV was used and a *k*‐point sampling set of 5 × 5 × 1 was tested to be converged. A force tolerance of 0.01 eV Å^−1^, energy tolerance of 5.0 × 10^−7^ eV per atom, and maximum displacement of 5.0 × 10^−4^ Å were considered. Gibbs free energy change (Δ*G*) of chemical reaction was calculated by the following equation: Δ*G* = Δ*E* + ΔZPE − *T*Δ*S*, where *E*, ZPE, *T*, and *S* denote the calculated total energy, zero‐point energy, temperature, and entropy, respectively. For the calculation of Δ*G* of other systems, including Ni–Mo–O, Fe–Mo–O, and Mo–O, the corresponding model was built by replacing Ni and/or Fe atoms by Mo atoms.

For the OER process, NiOOH, Fe‐doped NiOOH, and Fe, Mo‐codoped NiOOH were employed to model the Ni, Ni–Fe, and Ni–Fe–Mo system during OER, respectively. The atomic ratio of Ni:Fe:Mo in the Ni–Fe–Mo system was 80:10:3 (see the Supporting Information for the calculation detail). The surfaces of NiOOH, Fe‐NiOOH, and Fe, Mo‐NiOOH were cut along the (100) direction and the conventional four‐step mechanism (*—OH, *—O, *—OOH, and *+O_2_; * denotes the active site) was adopted to describe the OER process. The Gibbs free energy diagrams were estimated by the following equation: Δ*G_i_* = Δ*E_i_* + ΔZPE*_i_* − *T*Δ*S_i_* − *eU*, where *i* represents *OH, *O, or *OOH intermediates, *U* is the potential against normal hydrogen electrode (NHE) at standard conditions, and *e* is the transferred charge. Other parameters were the same with those of HER calculation.

## Conflict of Interest

The authors declare no conflict of interest.

## Supporting information

Supporting Information

Supplemental Video 1

Supplemental Video 2
