# Supplementary material for: Amorphous Ni–Fe–Mo Suboxides Coupled with Ni Network as Porous Nanoplate Array on Nickel Foam: A Highly Efficient and Durable Bifunctional Electrode for Overall Water Splitting
Source: Adv Sci (Weinh). 2020 Feb 5;7(7):1902034. doi: 10.1002/advs.201902034 (PMC7141049; doi:10.1002/advs.201902034)
Supplement: Supplementary file 1 — Supporting Information [file ADVS-7-1902034-s001.pdf]

Copyright WILEY-VCH Verlag GmbH & Co. KGaA, 69469 Weinheim, Germany, 2020.

ADVANCED SCIENCE
Open Access

Supporting Information

for *Adv. Sci.,* DOI: 10.1002/advs.201902034

Amorphous Ni–Fe–Mo Suboxides Coupled with Ni Network as Porous Nanoplate Array on Nickel Foam: A Highly Efficient and Durable Bifunctional Electrode for Overall Water Splitting

Yong-Ke Li, Geng Zhang, Wang-Ting Lu, and Fei-Fei Cao**

Copyright WILEY-VCH Verlag GmbH & Co. KGaA, 69469 Weinheim, Germany, 2020.

Supporting Information

Amorphous Ni-Fe-Mo Suboxides Coupled with Ni Network as Porous Nanoplate Array on Nickel Foam: A Highly Efficient and Durable Bifunctional Electrode for Overall Water Splitting

Yong-Ke Li,[‡] Geng Zhang,[‡,] Wang-Ting Lu and Fei-Fei Cao**

[‡]Y.-K. Li and G. Zhang contributed equally to this work.

Characterizations of NiFeMo/NF-Pre

NF-supported Ni-Fe LDH (Ni-Fe LDH/NF) was hydrothermally treated in a solution containing AHM. The color of electrode was changed from silver white of NF to yellow green of Ni-Fe LDH/NF and then to reddish brown of AHM-treated Ni-Fe LDH/NF (Figure S3). During AHM-treatment, more and more particles were observed on the surface of Ni-Fe LDH, and the Mo content gradually increases to 56.8% after 10 h (Figure S4), indicating that the particles may well be composed of compounds containing Mo element. The TEM image clearly shows that particles are deposited on the surface of Ni-Fe LDH nanosheet (Figure S5a). Specifically, Figure S5b demonstrates an interface between Ni-Fe LDH and two particles. The HRTEM image of the selected area in Figure S5c shows lattice fringes of 0.26 nm with an angle of 120°, which can be attributed to the (-100) and (1-10) plane of Ni-Fe LDH with the assistance of the FFT pattern.[1] The element mapping of AHM-treated Ni-Fe LDH provides an interesting image: most of Fe atoms are distributed in the particle instead of Ni-Fe LDH (Figure S6). On the basis of elemental mapping and line profile (Figure S6-S7), it can be concluded that particles are consisted of Ni, Mo, Fe and O element. The Raman spectra show that the peaks ascribed to the vibration of Ni-O (452 and 532 cm^{-1}) for Ni-Fe LDH/NF are weakened after AHM treatment; meanwhile, a series of Mo-O vibration modes at 288, 340,

818, 940 and 948 cm^{-1} appear (Figure S8).[2] The XPS spectra of AHM-treated Ni-Fe LDH/NF show that the Mo and Ni element exist in Mo^{6+} and Ni^{2+} valence, respectively (Figure S9). In addition, the line shape of the O K-edge XANES of AHM-treated Ni-Fe LDH/NF is like that of MoO_3, but quite different from that of NiO and Fe_2O_3 (Figure S10a); and a pre-edge peak at 20007 eV is observed in the Mo K-edge XANES (Figure S10b), which can be assignable to an inner-atomic 1s-4d electronic transition in the distorted MoO_6 octahedral unit, just like in MoO_3,[3] indicating that the particle deposited on Ni-Fe LDH should be an oxide with a structure similar with MoO_3.[4] The intense white line in the Ni K-edge XANES and pre-edge peak position (8329 eV) similar with that of NiO suggests the existence of Ni^{2+} species (Figure S10c).[5] As for Fe, the similar intensity of white line and the same location of pre-edge peak (7112 eV) in the Fe K-edge XANES with FeO (Figure S10d),[6] indicating Fe^{2+} ions reside in AHM-treated Ni-Fe LDH/NF. As a result, the particle is probably comprised of molybdate anion, Ni^{2+} cation and Fe^{2+} cation. Unfortunately, no PDF cards can be matched with the XRD pattern of AHM-treated Ni-Fe LDH when Ni, Fe and Mo element are selected at the same time. Instead, the XRD pattern (Figure S11) is close to that of $NiMo_3O_{19}·9H_2O$ and $H_{4.5}Mo_{5.25}O_{18}(H_2O)_{1.36}$, suggesting that the formed Ni-Fe-Mo-O compound may possess similar crystalline structure with these two compounds. Therefore, the AHM-treated Ni-Fe LDH/NF consists of Ni-Fe-Molybdate (NFM) and Ni-Fe_{trace} LDH (only trace of Fe left in LDH), and it is named NiFeMo/NF-Pre for clarity where Pre means it is the precursor of the final product.

The formation mechanism of NiFeMo/NF-Pre was then analyzed. We found that NFM failed to form when Ni-Fe LDH powder instead of Ni-Fe LDH/NF was treated in the same AHM solution (Figure S12). Moreover, if the substrate was changed from NF to carbon cloth, NFM cannot form on the surface of NiFe LDH as well (Figure S13). In contrast, if NF instead of Ni-Fe LDH/NF was used in the synthesis, a thick layer supported flower-like structure was generated on the NF substrate (Figure S14). Furthermore, the Mo content in NiFeMo/NF-Pre

can be tailored by either reaction duration (Figure S4e) or the AHM concentration (Figure S15). Therefore, it is believed that the combination of NF and Ni-Fe LDH nanosheet array is indispensable to obtain such special architecture of NiFeMo/NF-Pre. During synthesis, NF and Ni-Fe LDH will be etched in the acidic AHM solution (pH = 5.04), and then the produced Ni and Fe ions react with molybdate ions to form solid crystals which subsequently deposit on the surface of Ni-Fe LDH nanosheet.

The composition of $NiFeMoO_x$ suboxide and NiFeMo (oxy)hydroxide for the modeling in DFT calculation

The active material on the surface of NF was peeled off by sonication in the ethanol and the resulting precipitate was measured by EDS. The atomic ratio of Mo, Ni and Fe in the active material is 12.3: 8.4: 1 (Figure S16). Because the active material consists of $NiFeMoO_x$ suboxides and Ni nanoparticles, the composition of $NiFeMoO_x$ and the ratio of $NiFeMoO_x$ to metallic Ni is unable to be obtained by EDS, instead they can only be estimated by the help of XPS. In the XPS spectrum of Ni $2p_{3/2}$, the area ratio between Ni^{2+} peak and Ni^0 peak is considered as the ratio between Ni in Ni-Fe-Mo suboxide and Ni in metallic (Figure 2a in the main text), and the result is 11.8: 1; furthermore, the atomic ratio of Mo: Ni: Fe in the Ni-Fe-Mo suboxide is 12.3: 7.7: 1.

According to physical characterizations, the structure of $NiFeMoO_x$ is based on MoO_3, and part of Mo atoms are replaced by Ni or Fe, and part of O atoms are removed to create O vacancies. Because Ni and Fe atoms are in +2 valence and Mo in +5 and +6 valence in $NiFeMoO_x$ suboxide, the atomic ratio of O in the suboxide is determined by the content of Mo^{5+}. According to the Mo 3d XPS spectrum of Ni/$NiFeMoO_x$/NF (Figure 2b in the main text), the atomic ratio between Mo^{5+} and Mo^{6+} is 59.2: 40.8. As a result, the atomic ratio of Ni:Fe:Mo:O in the model of $NiFeMoO_x$ is 8:1:12:42 after approximation.

For the Ni-Fe-Mo system used in the OER process, the content of Mo is determined by the XPS spectra after long-term OER process. The Mo content measured by XPS is decreased from 73 at.% to 1.7 at.% (on the basis of total metal atoms, Figure S42). The Ni:Fe ratio in the Ni-Fe-Mo (oxy)hydroxide is the same with that of $NiFeMoO_x$. Thus, the Ni:Fe:Mo atomic ratio in NiFeMo (oxy)hydroxide is estimated to be 80:10:3.

Table S1 The comparison of catalytic performances for HER in 1 M KOH between Ni/NiFeMoO$_x$/NF and other electrodes reported in the literature.

Electrode	η_{10} mV	η_{100} mV	Tafel Slope mV dec^{-1}	Mass Loading mg cm^{-2}	C_{dl} mF cm^{-2}	Morphology	Ref.
Ni/NiFeMoO$_x$/NF	**22**	**117**	**76**	**1.8**	**442**	**Nanoplate Assembled by Nanoparticles**	**This work**
MoO$_x$/Ni$_3$S$_2$/NF	116	224	90	12	145	Hollow Microsphere	[7]
MoS$_2$/Ni$_3$S$_2$ heterostructures/NF	110	n.a.	83	9.7	15.6	Nanosheet&Nanoparticle	[8]
NiMoN/NF-450	22	>100	101	n.a.	5.5	Nanowire	[9]
MoS$_2$–Ni$_3$S$_2$ HNRs/NF	98	191	61	13	121.3	Nanorod	[10]
NiFeMo/NF	45	~110	n.a.	1.6	75	Film	[11]
Am FePO$_4$/NF	123	~250	104	0.285	n.a.	Nanosheet	[12]
MoS$_2$/FNS/FeNi	122	~300	45	0.153	n.a.	Nanosheet	[13]
Porous MoO$_2$/NF	27	n.a.	41	3.4	422	Nanosheet	[14]
NFL MoO$_2$/NF	55	n.a.	66	4.5	n.a.	Nanoflower	[15]
MoNi$_4$ networks/NF	28	n.a.	36	1.09	374	Nanosheet	[16]
Ni-Mo nanosheets/NF	35	136	45	0.8	n.a.	Nanosheet	[17]
NC/NiMo/NiMoO$_x$/NF	29	~105	46	20	90.8	Nanowire	[18]
Ni$_{2(1-x)}$Mo$_{2x}$P/NF	72	162	46	n.a.	51.2	Nanowire	[19]
Cu@CoS$_x$/CF	134	267	61	3.9	77.5	Film	[20]
nest-like NiCoP/CC	62	158	68	2	51.5	Nanowire	[21]
MoP NWAs/CFP	52	~100	40	n.a.	104	Nanowire	[22]
Cu@NiFe LDH/CF	116	192	59	n.a.	59.8	Nanowire&Nanosheet	[23]
NFN-MOF/NF	87	~210	35	0.6	n.a.	Nanosheet	[24]
NiFeRu LDH/NF	29	~120	31	1.2	5.9	Nanosheet	[25]
Co$_1$Mn$_1$CH/NF	180	328	n.a.	5.6	380.2	Nanosheet	[26]
Ni-FeP/TiN/CC	75	~195	73	n.a.	33.7	Nanowire	[27]
Ni$_3$N-VN/NF	64	218	37	n.a.	184	Nanosheet&Nanoparticle	[28]
FeS/IF	n.a.	243	77	n.a.	n.a.	Nanosheet&Nanoparticle	[29]
NF@Ni/C-600	37	~200	57	7.3	95	Nanosheet	[30]
Ni$_3$(S$_{0.25}$Se$_{0.75}$)$_2$@NiOOH/NF-8	102	~240	47	n.a.	23.2	Dendrite	[31]

Notes: η_{10} and η_{100} means overpotential at 10 and 100 mA cm^{-2}, respectivley. C_{dl} stands for double layer capacitance.

Table S2 The comparison of catalytic performances for OER in 1 M KOH between Ni/NiFeMoO$_x$/NF and and other electrodes reported in the literature.

Electrode	η_{10} mV	η_{100} mV	Tafel slope mV dec^{-1}	Mass Loading mg cm^{-2}	C_{dl} mF cm^{-2}	Morphology	Ref.
**Ni/NiFeMoO$_x$/NF**	**255**	**289**	**35**	**1.8**	**442**	**Nanoplate Assembled by Nanoparticles**	**This work**
MoO$_x$/Ni$_3$S$_2$/NF	136	310	n.a.	See Table S1			[7]
MoS$_2$/Ni$_3$S$_2$ heterostructures/NF	218	~290	88	See Table S1			[8]
MoS$_2$−Ni$_3$S$_2$ HNRs/NF	249	341	57	See Table S1			[10]
CP/CTs/Co-S	306	n.a.	72	0.32	103.7	Nanotube&Nanosheet	[32]
NiFeMo LDH/NF	n.a.	~276	n.a.	n.a.	n.a.	Nanosheet	[33]
NiFeMo/NF	238	290	35	See Table S1			[11]
MoS$_2$/FNS/FeNi	204	~240	28.1	See Table S1			[13]
NC/NiMo/NiMoO$_x$/NF	284	~380	111	See Table S1			[18]
NiMoN/NF-450	230	~370	116	See Table S1			[9]
P-CoMoS/CC	260	~460	70.2	2.19	34.87	Nanosheet	[34]
Fe-Mn-O NSs/CC	273	397	64	1.6	116.3	Nanosheet	[35]
Cu@CoS$_x$/CF	160	310	n.a.	See Table S1			[20]
NiCoON(1:2) NSAs/NF	247	n.a.	35	0.6	141	Nanosheet	[36]
NiOOH–NiCr$_2$O$_4$/NF	n.a.	~340	104	n.a.	102	Nanosheet	[37]
Co$_{0.93}$Ni$_{0.07}$P$_3$ NAs/CC	n.a.	~360	83.7	1.05	80	Nanowire	[38]
nest-like NiCoP/CC	242	330	64.2	See Table S1			[21]
NC/CuCo/CuCoO$_x$/NF	198	294	88	1.5	76.8	Nanowire	[39]
Am FePO$_4$/NF	218	~260	42.7	See Table S1			[12]
Cu@NiFe LDH/CF	199	281	27.8	See Table S1			[23]
NFN-MOF/NF	240	~310	58.8	See Table S1			[24]
Co-Ni-Se/C/NF	n.a.	~330	63	n.a.	80.6	Nanosheet&Nanoparticle	[40]
NiFeRu LDH/NF	225	~250	n.a.	See Table S1			[25]
Co$_1$Mn$_1$CH/NF	n.a.	349	n.a.	See Table S1			[26]
Ni$_2$P-VP$_2$/NF	n.a.	398	49	See Table S1			[28]
FeS/IF	238	537	n.a.	See Table S1			[29]
NF@Ni/C-600	265	470	54	See Table S1			[30]
Ni$_3$(S$_{0.25}$Se$_{0.75}$)$_2$@NiOOH/NF-8	n.a.	340	48	See Table S1			[31]

Notes: η_{10} and η_{100} means overpotential at 10 and 100 mA cm^{-2}, respectivley. C_{dl} stands for double layer capacitance.

Table S3 The comparison of catalytic performances for overall water splitting in 1 M KOH between Ni/NiFeMoO$_x$/NF and other bifunctional electrodes reported in the literature.

Electrode	$U_{@10\ mA\ cm-2}$	$U_{@100\ mA\ cm-2}$	Durability	Ref.
**Ni/NiFeMoO$_x$/NF**	**1.50**	**1.63**	**100 h@500 mA cm^{-2}**	**This Work**
NiMoN/NF-450	1.507	~1.67	34 h@370 mA cm^{-2}	[9]
Porous MoO$_2$/NF	1.53	n.a.	24 h@10 mA cm^{-2}	[14]
MoO$_x$/Ni$_3$S$_2$/NF	1.45	1.71	100 h@1.5 V(~10 mA cm^{-2})	[7]
MoS$_2$/Ni$_3$S$_2$ heterostructures/NF	1.56	>1.65	10 h@10 mA cm^{-2}	[8]
MoS$_2$-Ni$_3$S$_2$ HNRs/NF	1.50	>1.65	45 h@1.53 V(~15 mA cm^{-2})	[10]
NC/NiMo/NiMoO$_x$/NF	1.57	>1.75	60 h@1.57, 1.65 and 1.75 V(< 80 mA cm^{-2})	[18]
P-CoMoS/CC	1.54	>2.0	100 h@10 mA cm^{-2}	[34]
NiFeMo/NF	1.45	1.81	50 h@1.6 V(~30 mA cm^{-2})	[11]
FeP/Ni$_2$P/NF	1.42	1.60	40 h@500 mA cm^{-2}	[41]
Cu@CoS$_x$/CF	1.50	1.80	200 h@100 mA cm^{-2}	[20]
2-cycle NiFeO$_x$/CFP	1.51	1.73	200 h@10 mA cm^{-2}	[42]
nest-like NiCoP/CC	1.52	~1.76	11 h@100 mA cm^{-2}	[21]
NC/CuCo/CuCoO$_x$/NF	1.53	n.a.	100 h@50 mA cm^{-2}	[39]
Am FePO$_4$/NF	1.54	1.72	15 h@10 mA cm^{-2}	[12]
Cu@NiFe LDH/CF	1.54	1.69	24 h@100 mA cm^{-2}	[23]
Fe$_{17.5\%}$-Ni$_3$S$_2$/NF	1.54	1.70	10 h@~20 mA cm^{-2}	[43]
FeNi-N/CFC	1.55	~1.75	60 h@1.8 V(362.1-392.8 mA cm^{-2})	[44]
NFN-MOF/NF	1.56	~1.73	30 h@500 mA cm^{-2}	[24]
NiFe$_2$O$_4$/NiFe LDH	1.535	~1.75	20 h@500 mA cm^{-2}	[45]
Co-Ni-Se/C/NF	1.60	~1.88	100 h@50 mA cm^{-2}	[40]
NiFeRu LDH/NF	1.52	~1.70	10 h@10 mA cm^{-2}	[25]
Co$_1$Mn$_1$CH/NF	1.68	1.78	14 h@10 mA cm^{-2}	[26]
(Ni$_{0.33}$Fe$_{0.67}$)$_2$P/NF	1.49	1.72	11 h@~100 mA cm^{-2}	[46]
CP/CTs/Co-S	1.743	n.a.	2 h@10 mA cm^{-2}	[32]
Ni-P/NF	1.64	2.05	1000 h@20 mA cm^{-2}	[47]
SCFP-NF	1.66	>1.8	650 h@10 mA cm^{-2}	[48]
Ni@Co-Ni-P	n.a.	1.93	3000 h@100 mA cm^{-2}	[49]
FeS/IF	1.65	1.80	50 h@~10 mA cm^{-2}	[29]
NF@Ni/C-600	n.a.	~1.88	70 h@~36 mA cm^{-2}	[30]
Ni$_3$(S$_{0.25}$Se$_{0.75}$)$_2$@NiOOH/NF-8	1.55	~1.90	36 h@10-90 mA cm^{-2}	[31]

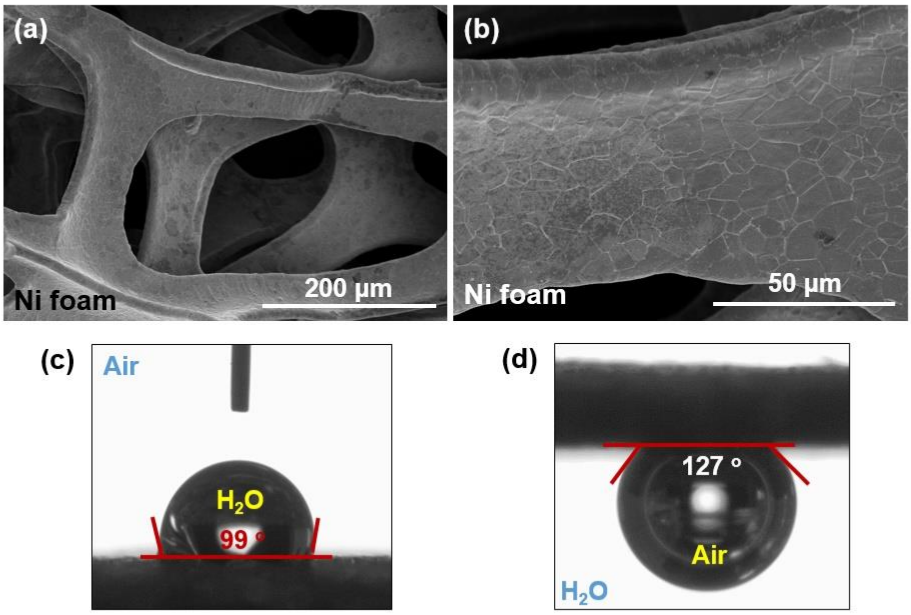

Figure S1 (a, b) SEM images of Ni foam (NF). (c) Water contact angle on the surface of NF in the air. (d) Air bubble contact angle on the surface of NF under water.

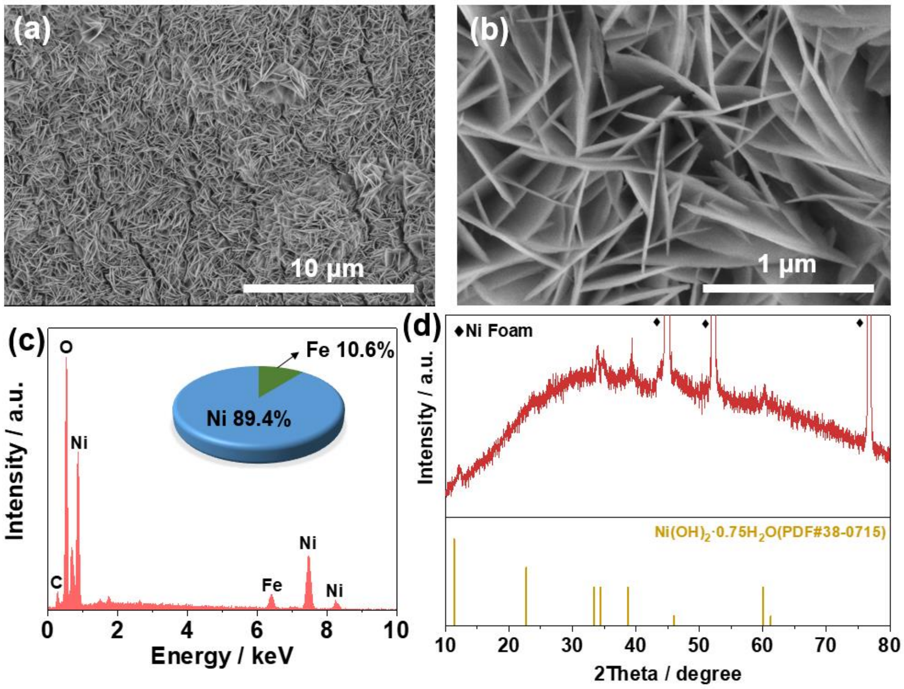

Figure S2 (a, b) SEM images, (c) EDS spectrum and (d) XRD pattern of Ni-Fe LDH/NF.

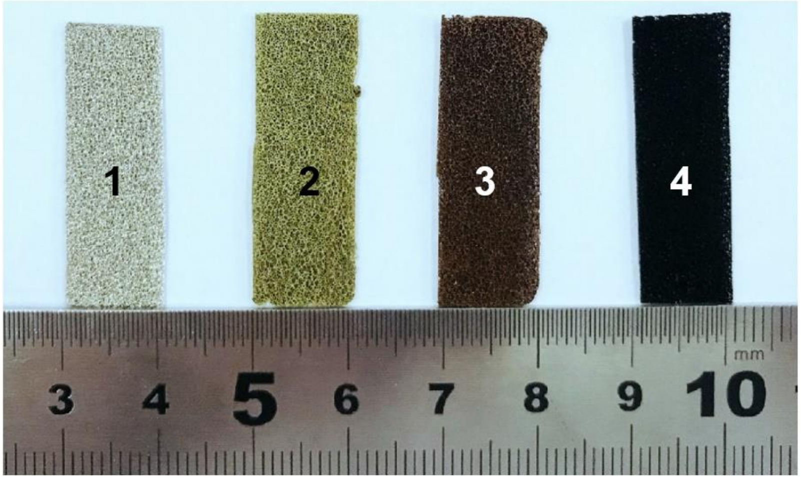

Figure S3 Photographs of (1) NF, (2) Ni-Fe LDH/NF, (3) NiFeMo/NF-Pre and (4) Ni/NiFeMoO_x/NF.

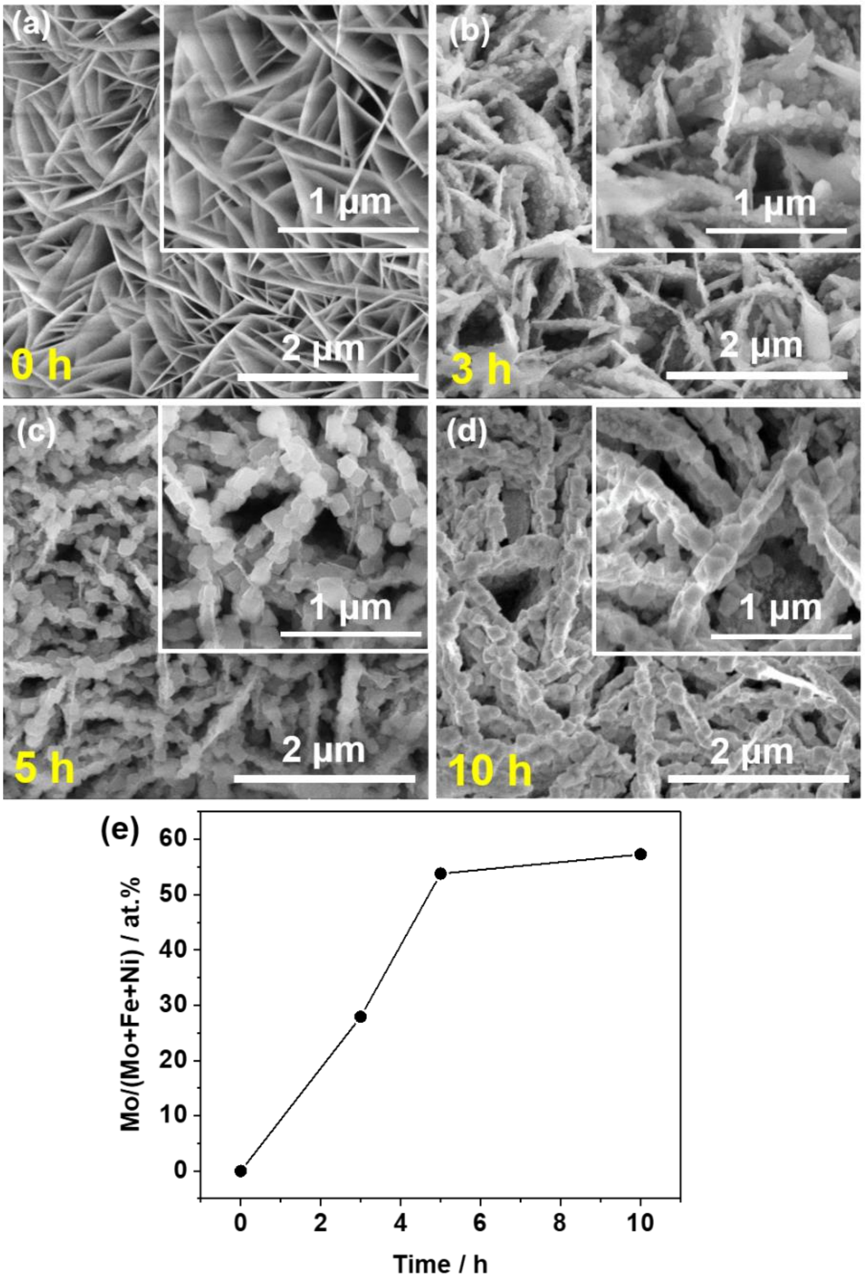

Figure S4 (a-d) SEM images of NiFeMo/NF-Pre obtained at various reaction duration. (e) The relationship of Mo content in NiFeMo/NF-Pre with the increase of reaction time.

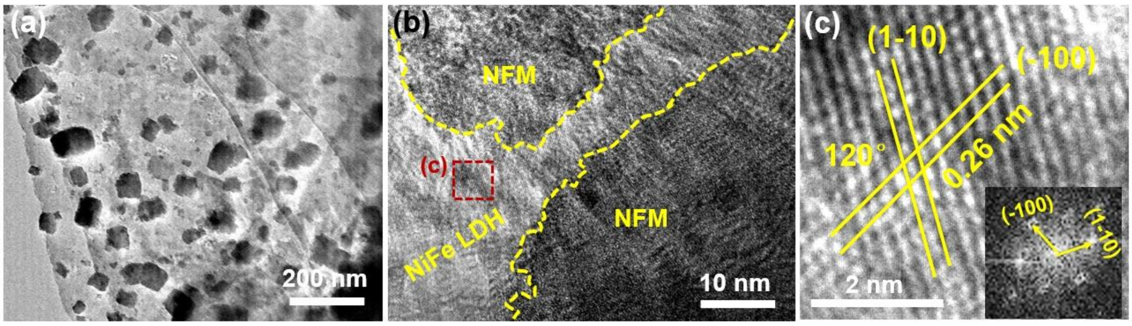

Figure S5 (a) TEM and (b, c) HRTEM images of NiFeMo/NF-Pre. NFM denotes Ni-Fe-Molybdate.

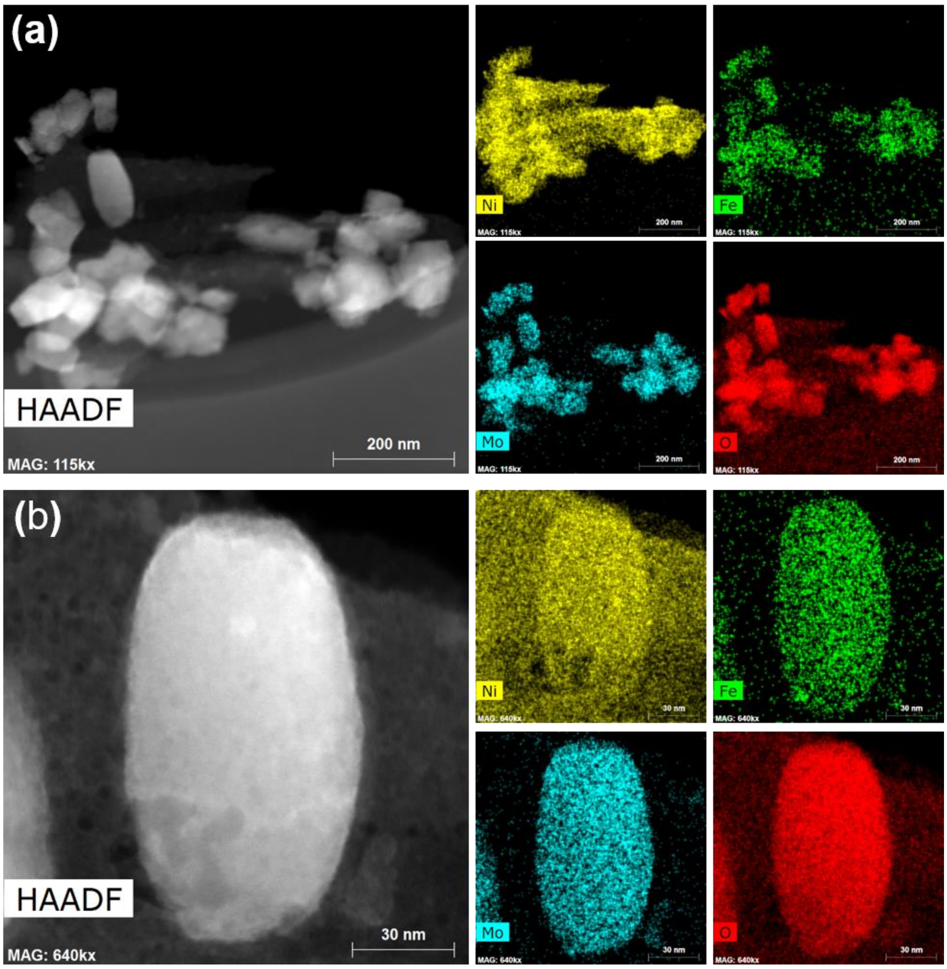

Figure S6 HAADF-STEM images and elemental mapping of NiFeMo/NF-Pre at (a) low and (b) high magnification.

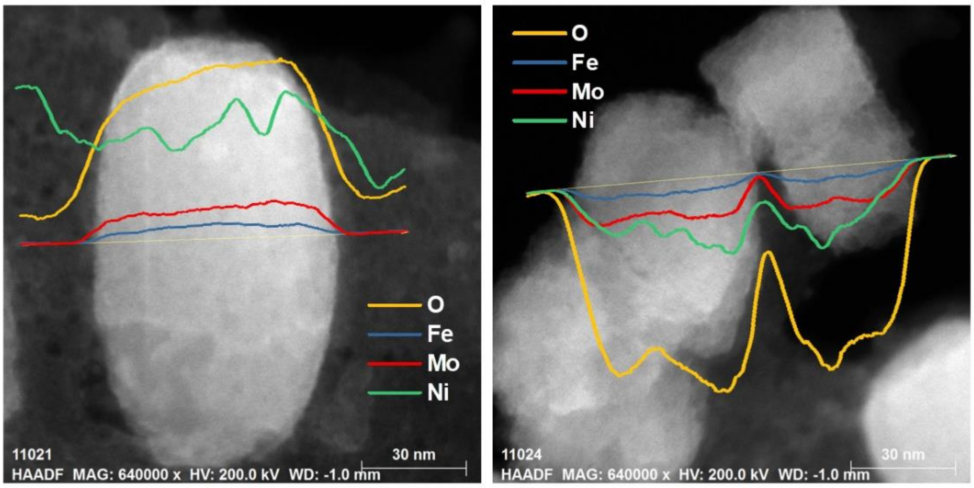

Figure S7 HAADF-STEM images and elemental line profiles of NiFeMo/NF-Pre.

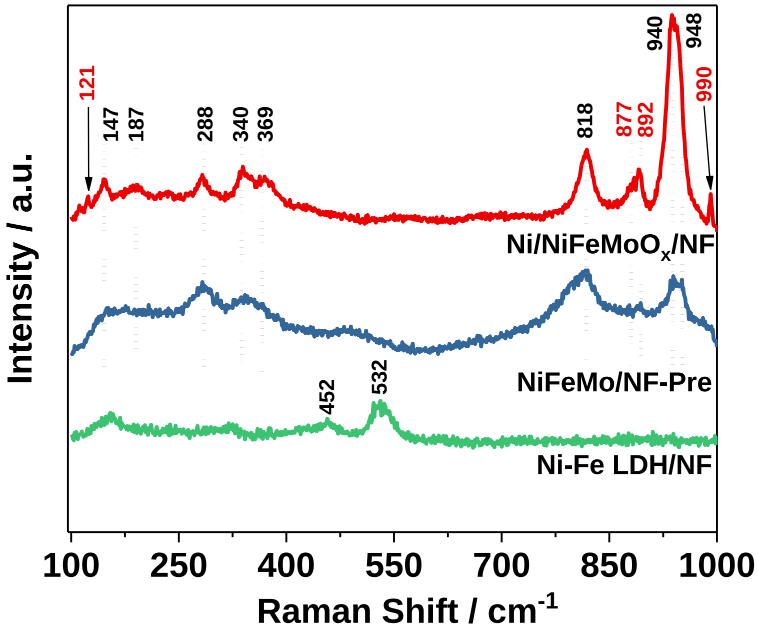

Figure S8 Raman spectra of Ni-Fe LDH/NF and NiFeMo/NF-Pre.

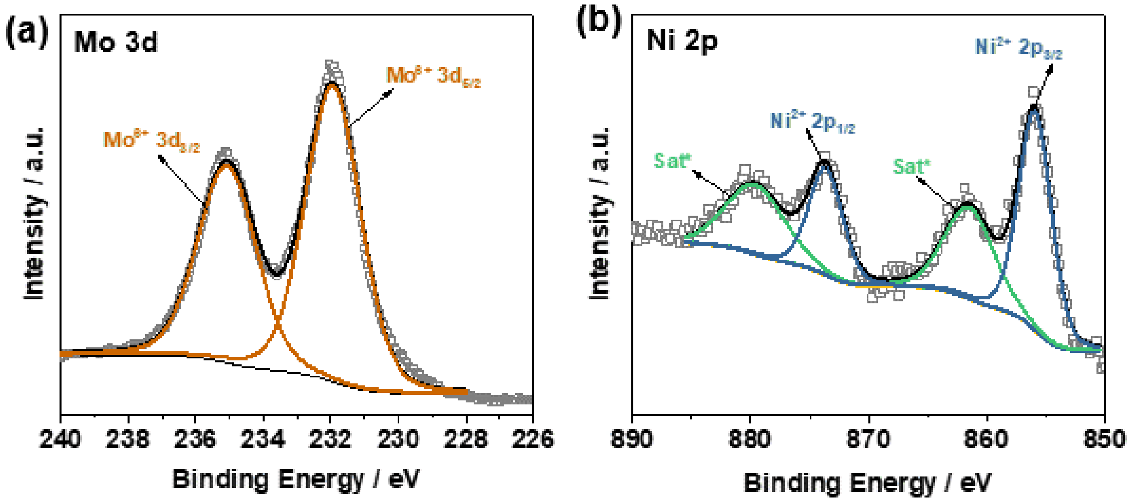

Figure S9 (a) Mo 3d and (b) Ni 2p XPS spectra of NiFeMo/NF-Pre.

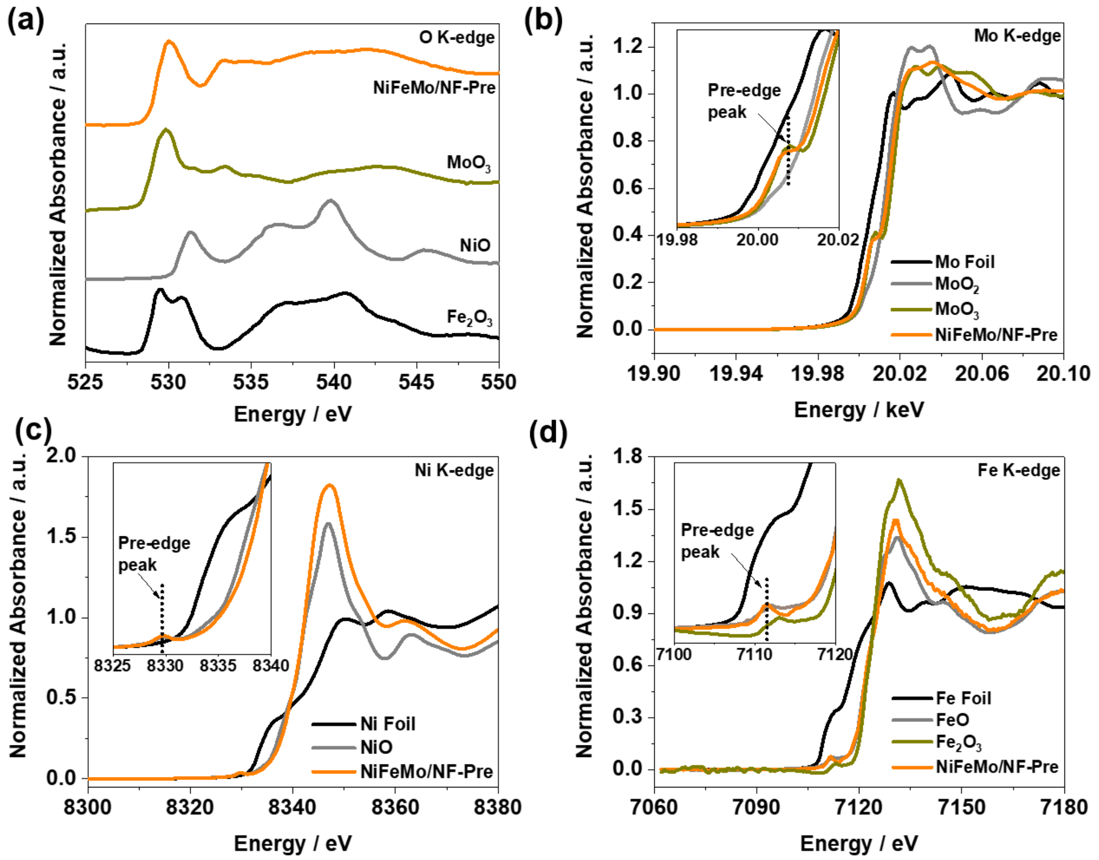

Figure S10 XANES spectra at the (a) O, (b) Mo, (c) Ni and (d) Fe K-edge for NiFeMo/NF-Pre and referenced materials.

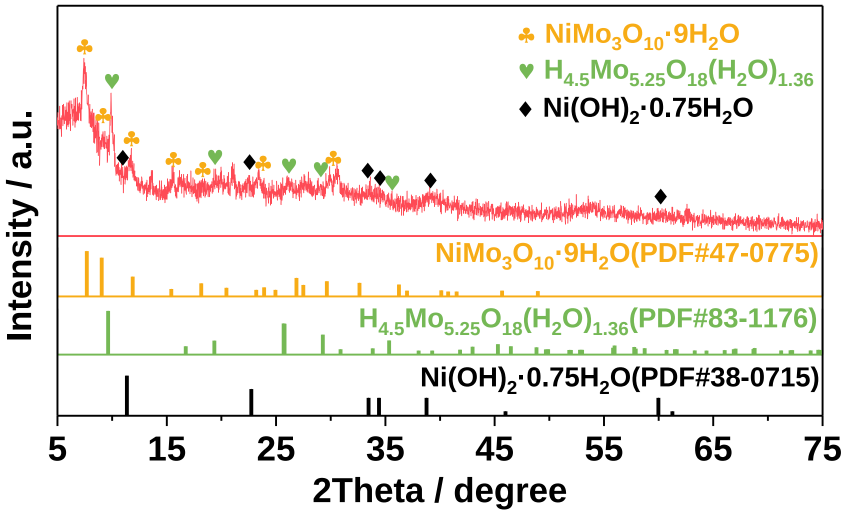

Figure S11 XRD pattern of NiFeMo/NF-Pre peeled off from the electrode.

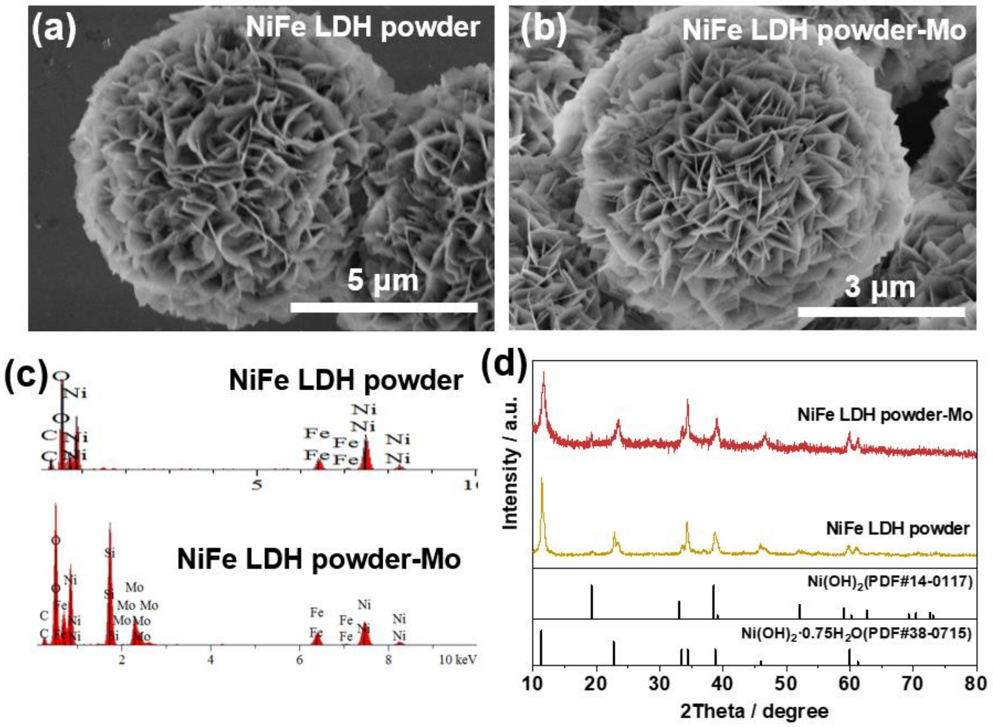

Figure S12 (a, b) SEM images, (c) EDS spectra and (d) XRD patterns of Ni-Fe LDH powder and the AHM-treated Ni-Fe LDH powder (named as Ni-Fe LDH-Mo).

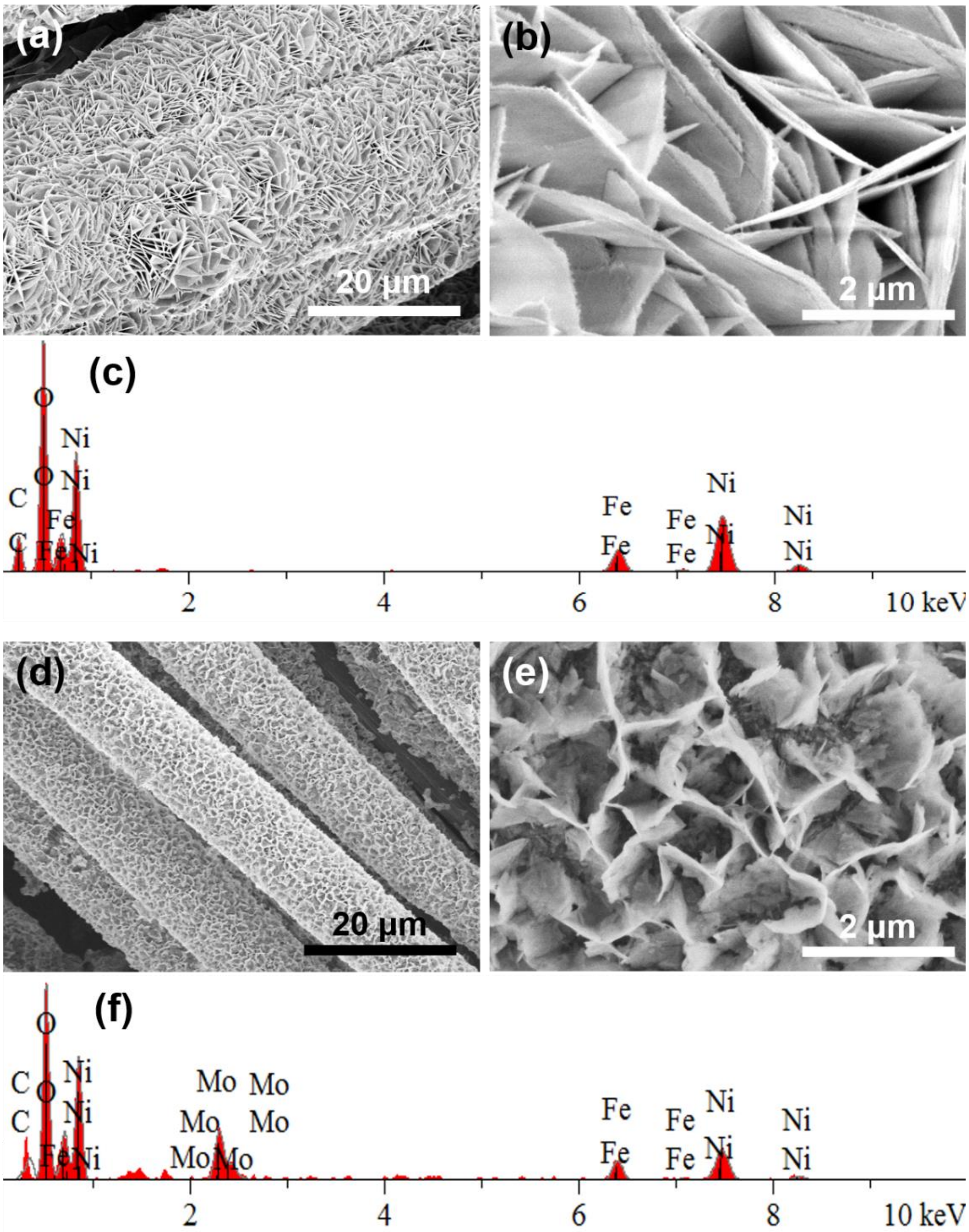

Figure S13 (a, b) SEM images and (c) EDS spectrum of Ni-Fe LDH/CC. (d, e) SEM images and (f) EDS spectrum of AHM-treated Ni-Fe LDH/CC.

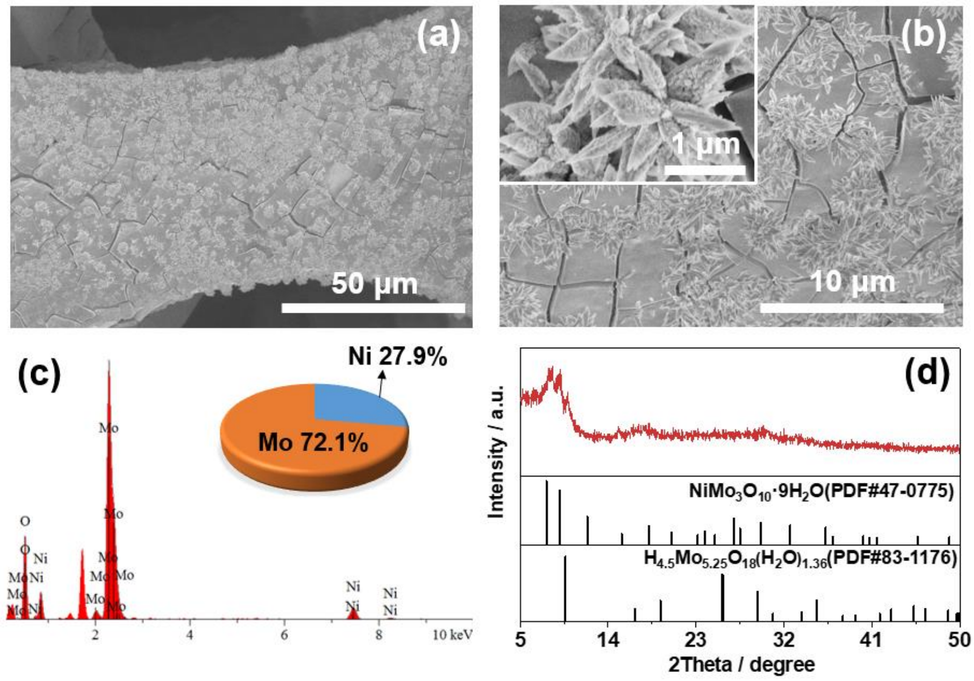

Figure S14 (a, b) SEM images, (c) EDS spectrum and (d) XRD pattern of NF-Mo electrode.

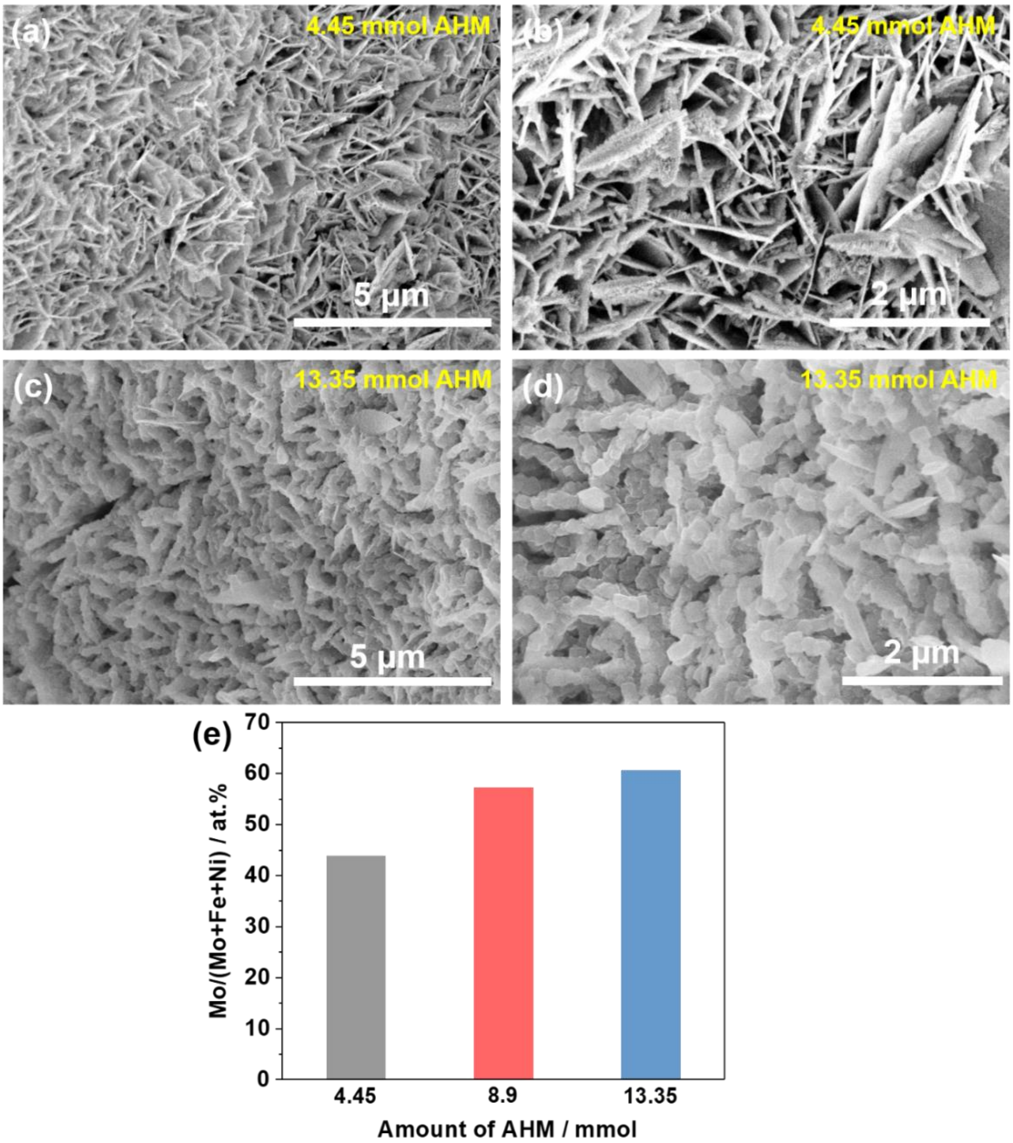

Figure S15 (a, b) SEM images of NiFeMo/NF-Pre electrode obtained at the amount of AHM of 4.45 mmol. (c, d) SEM images of NiFeMo/NF-Pre electrode obtained at the amount of AHM of 13.35 mmol. (e) Mo content in the NiFeMo/NF-Pre electrode obtained at different amount of AHM.

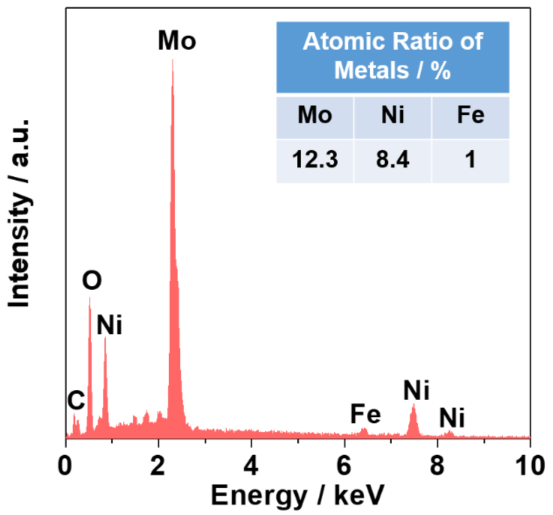

Figure S16 EDS spectrum of Ni/NiFeMoO$_x$/NF which is peeled off from the electrode by ultrasonication in ethanol.

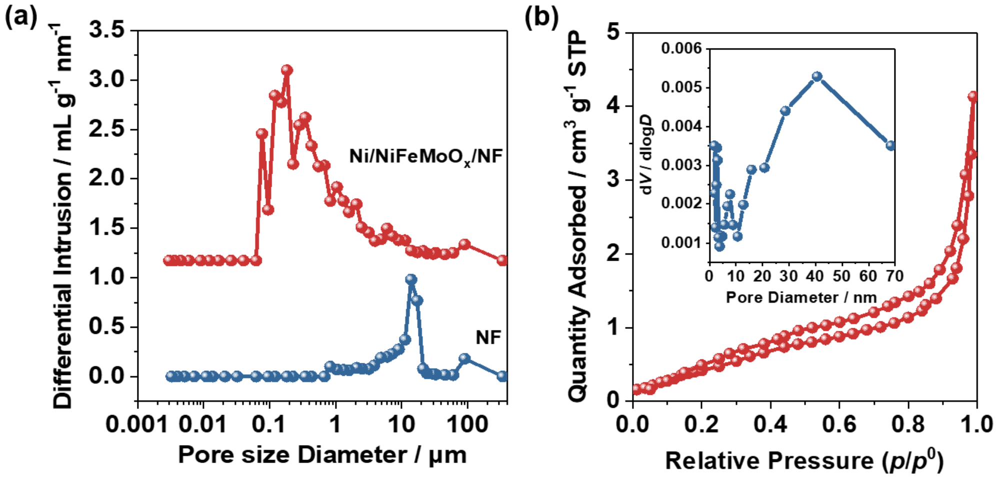

Figure S17 (a) Pore size distribution of NF and Ni/NiFeMoO$_x$/NF derived from mercury intrusion analysis. (b) N_2-sorption isotherms and (inset) pore size distribution for Ni/NiFeMoO$_x$/NF.

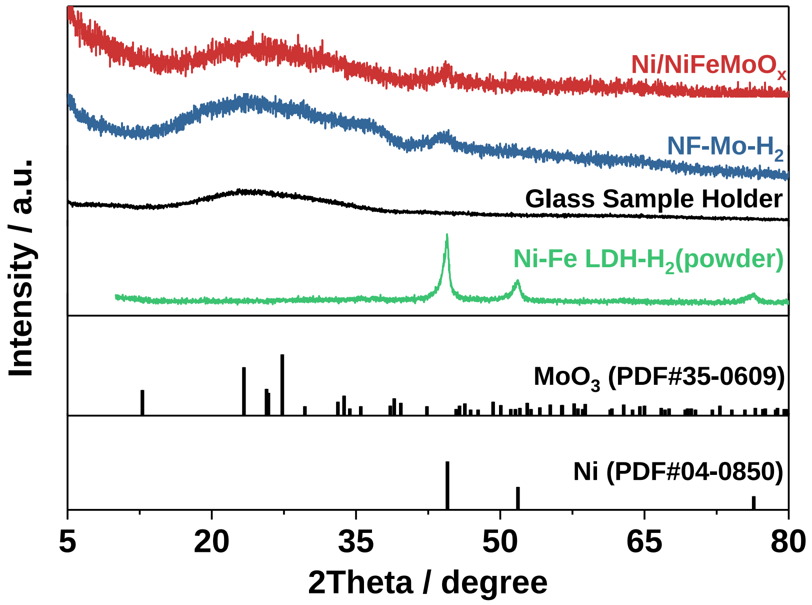

Figure S18 XRD patterns of Ni/NiFeMoO$_x$/NF and NF-Mo-H$_2$ which are peeled off from the electrode by ultrasonication in ethanol. The XRD pattern of reduced Ni-Fe LDH powder by H$_2$ is also displayed.

The reduced Ni-Fe LDH powder shows a series of diffraction peaks ascribed to face-centered-cubic (FCC) metallic Ni (PDF#04-0850), where the reduced Fe atoms will be alloyed with Ni due to the low content of Fe. In order to characterize the phase of Ni/NiFeMoO$_x$/NF, the deposit is peeled off from the NF substrate by sonication in ethanol followed by XRD analysis to avoid the interference of NF. The peaks ascribed to Ni-Fe LDH, $NiMo_3O_{19}\cdot 9H_2O$ and $H_{4.5}Mo_{5.25}O_{18}(H_2O)_{1.36}$ disappeared after reduction (Figure S11); instead, only a distinct peak centered at ~44° is observed. The peak at ~44° can be assigned to metallic Ni transformed from Ni-Fe$_{trace}$ LDH in NiFeMo/NF-Pre.

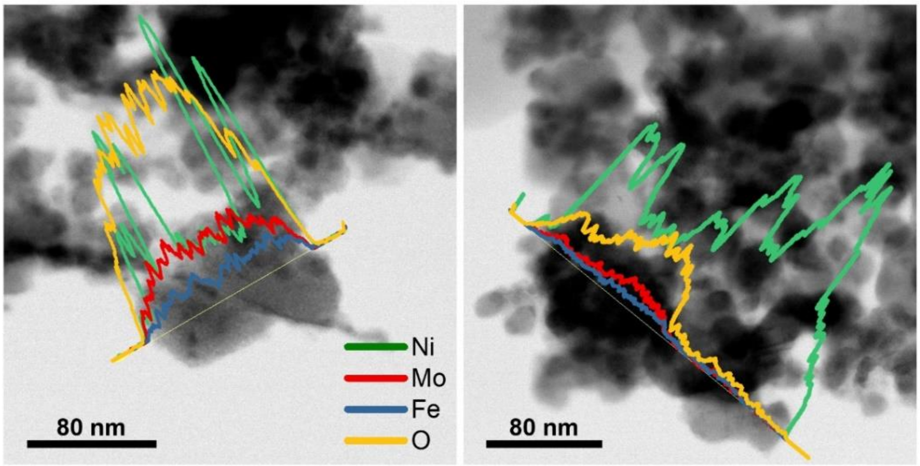

Figure S19 STEM images and elemental line profiles of Ni/NiFeMoO$_x$/NF at different locations.

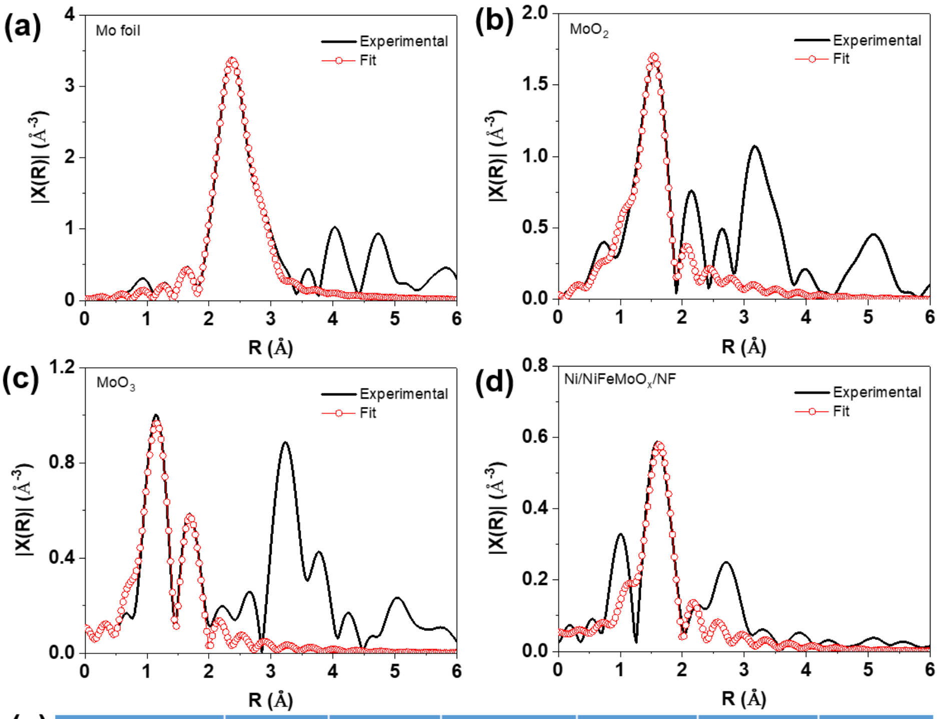

(e)

	Path	N	ΔE(eV)	100×R(Å)	$10^3\times\sigma^2$(Å^2)	R-factor
**Mo foil**	Mo-Mo1	6	-8.50(0.98)	217.6(0.44)	3.00(0.52)	0.006
	Mo-Mo2	6	-8.50(0.98)	313.2(0.64)	3.99(0.69)	
**MoO_2**	Mo-O	6	-0.15(2.88)	201.1(1.60)	2.24(1.92)	0.010
**MoO_3**	Mo-O1	3	-7.99(3.03)	163.2(1.62)	0.00(200)	0.029
	Mo-O2	3	-7.99(3.03)	225.9(1.62)	0.00(3.01)	
**Ni/NiFeMoO$_x$/NF**	Mo-O	3.42(0.79)	8.31(2.62)	208.7(1.98)	0.11(2.60)	0.037

Figure S20 (a-d) FT-EXAFS spectra and corresponding fitted line for (a) Mo foil, (b) MoO_2, (c) MoO_3 and (d) Ni/NiFeMoO$_x$/NF. (e) Parameters extracted from the fits of EXAFS data.

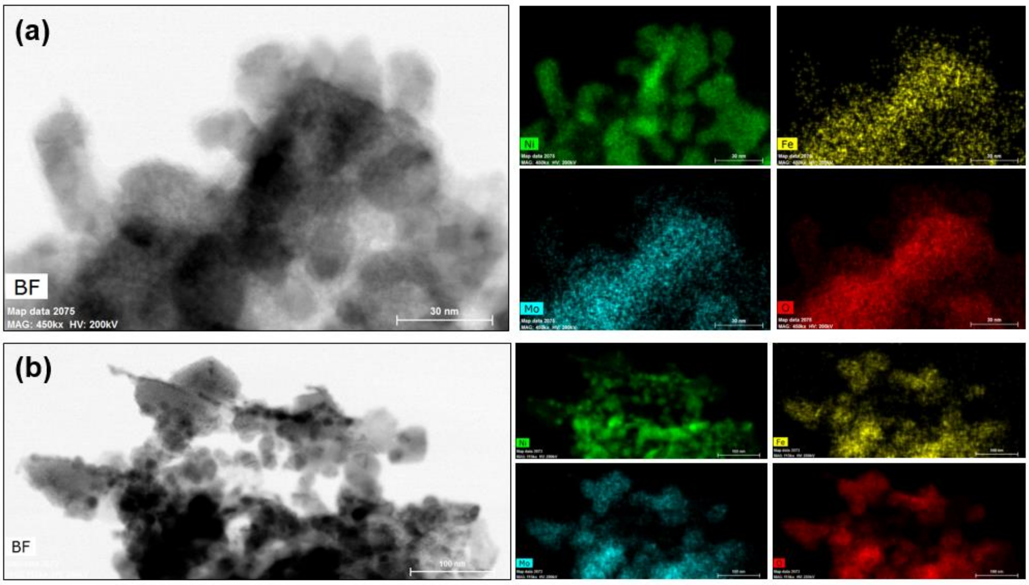

Figure S21 STEM images and elemental mapping of Ni/NiFeMoO$_x$/NF at different locations.

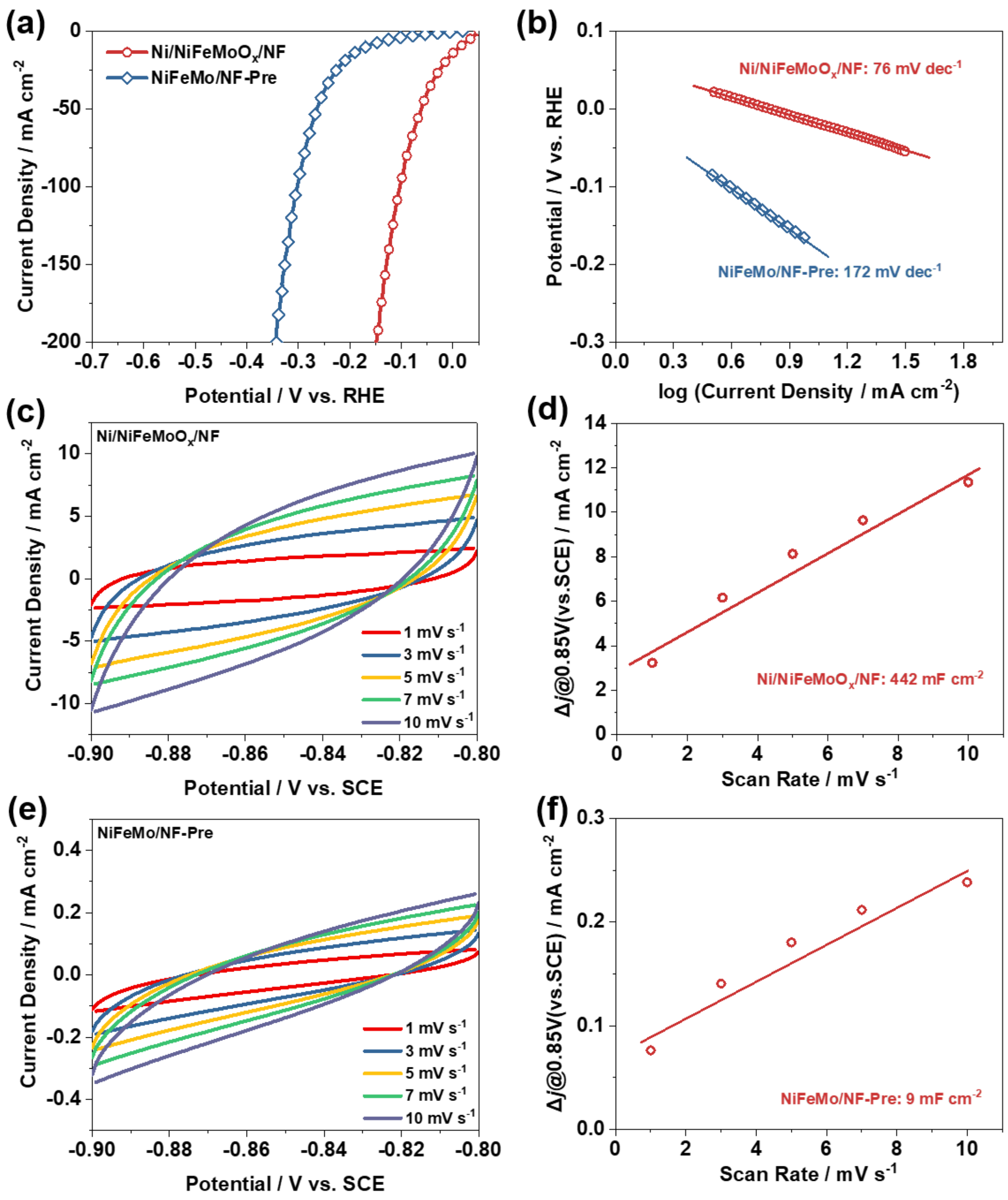

Figure S22 (a) *iR*-Corrected LSV curves and (b) Tafel plots of NiFeMo/NF-Pre and Ni/NiFeMoO$_x$/NF for HER. (c) CV curves and (d) double layer capacitance (C_{dl}) for NiFeMo/NF-Pre. (e) CV curves and (f) C_{dl} for Ni/NiFeMoO$_x$/NF. The value of C_{dl} is half of the plot slope.

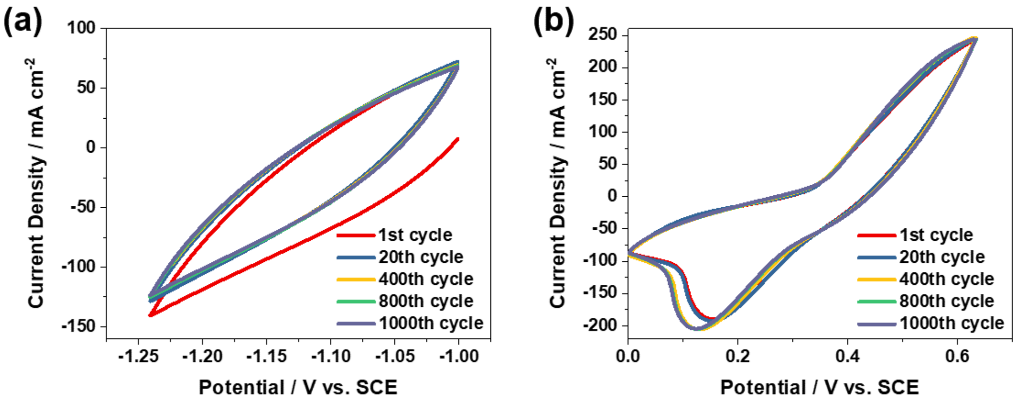

Figure S23 Durability testing process under CV cycling conditions for (a) HER and (b) OER.

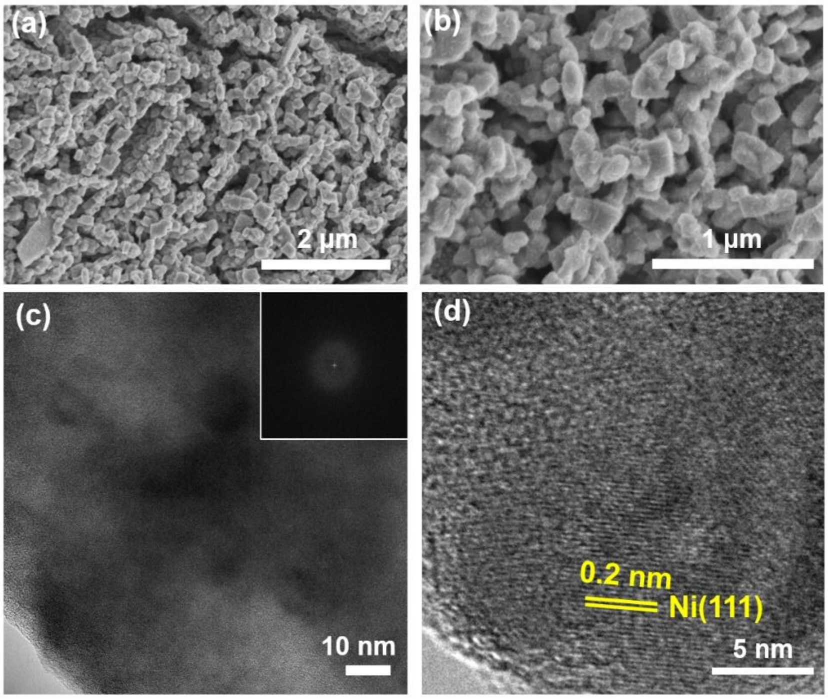

Figure S24 (a, b) SEM images and (c, d) TEM images of Ni/NiFeMoO$_x$/NF electrode after hydrogen evolution for 24 h. The inset in panel (c) shows its corresponding FFT pattern.

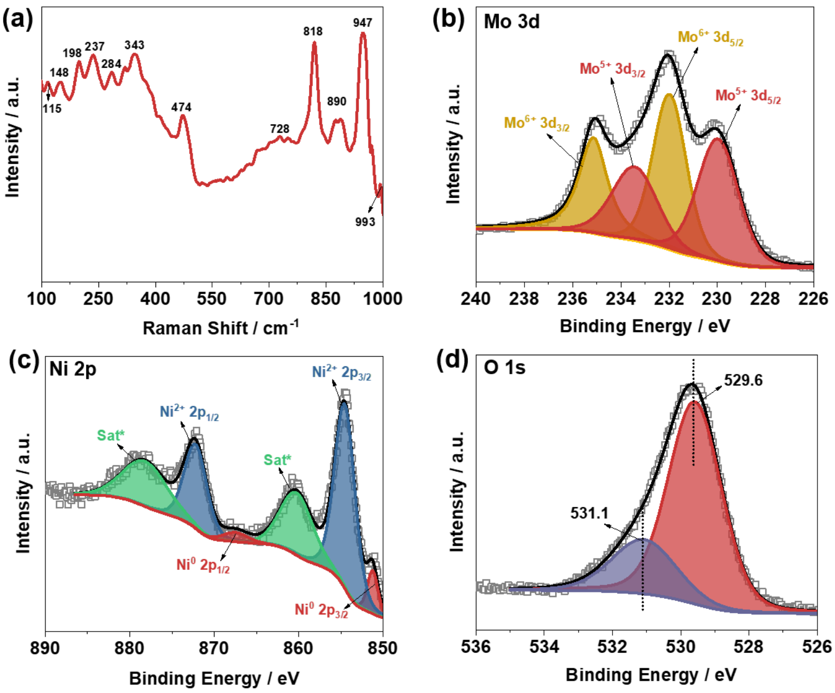

Figure S25 (a) Raman spectrum and (b-d) XPS spectra of Ni/NiFeMoO$_x$/NF electrode after hydrogen evolution for 24 h.

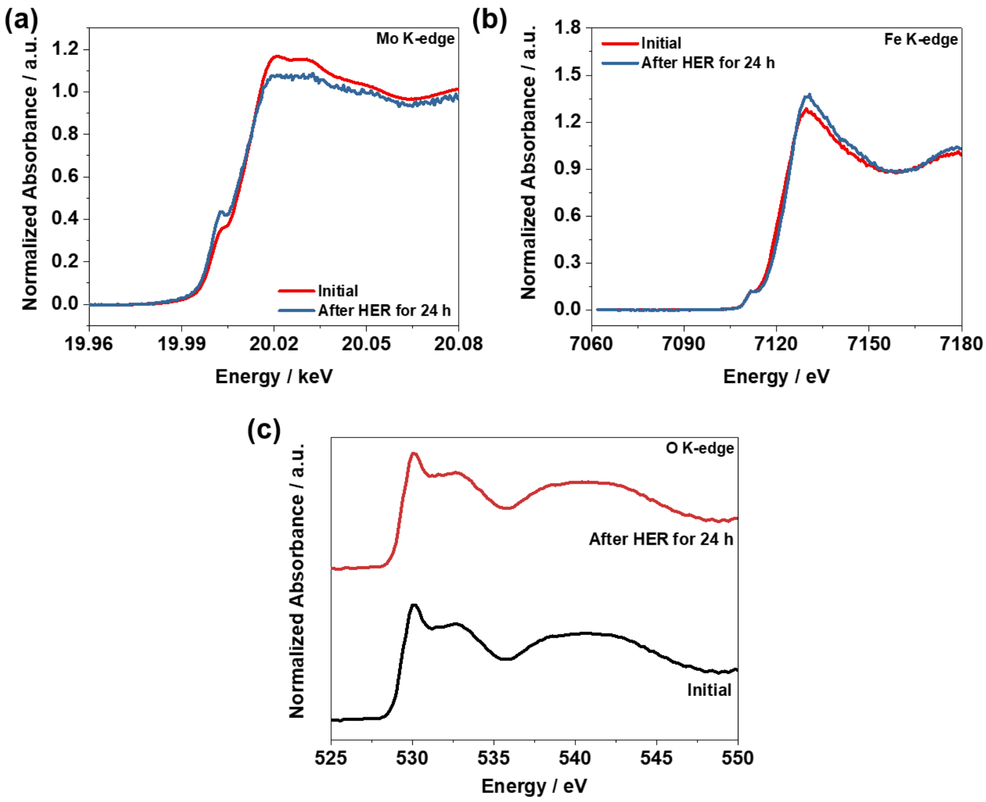

Figure S26 XANES spectra at (a) Mo, (b) Fe and (c) O K-edge for Ni/NiFeMoO$_x$/NF after hydrogen evolution for 24 h.

After long-term hydrogen evolution, the locations of Raman peaks (Figure S25a) are almost the same with that of the pristine electrode (Figure S8).The Mo^{5+} signal (Figure S25b), Ni^{2+} signal (Figure S25c) and negative shift of the lattice O (Figure S25d) are still observed by XPS. Little changes ocurr on the XANES of Mo, Fe and O elements (Figure S26). In addition, the porous array morphology is well maintained (Figure S24a-S24b). HRTEM images demonstrate that the $NiFeMoO_x$ suboxide (Figure S24c) and Ni nanoparticle (Figure S24d) exists in an amorphous and crystalline structure, respectively. All the above results indicate that amorphous $NiFeMoO_x$ suboxides are left.

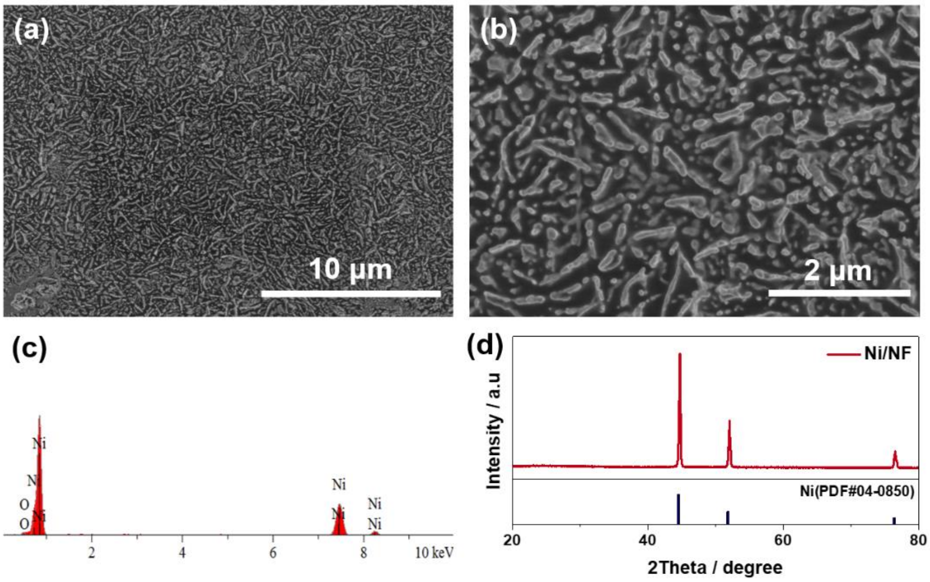

Figure S27 (a-b) SEM images, (c) EDS spectrum and (d) XRD pattern of Ni/NF.

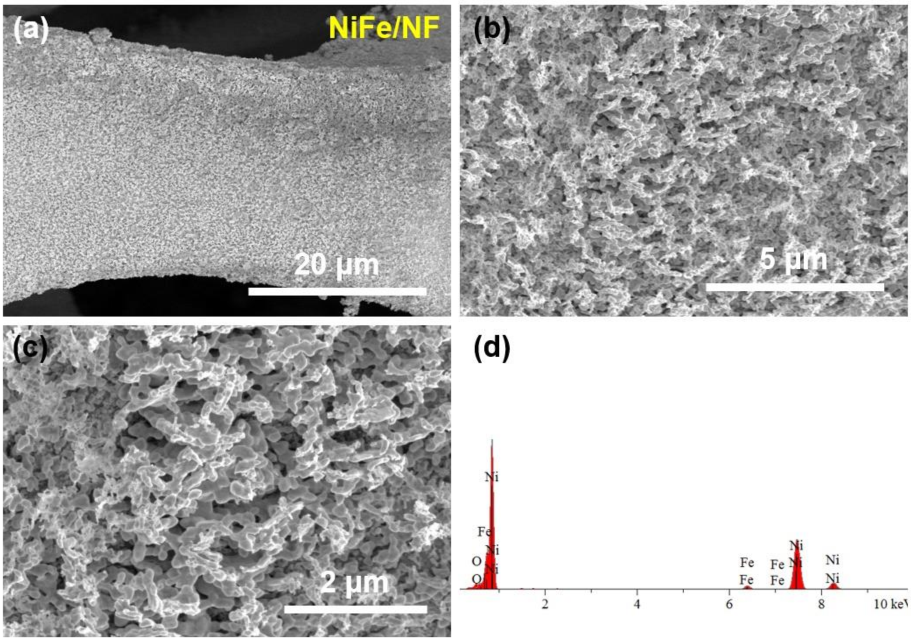

Figure S28 (a-c) SEM images and (d) EDS spectrum of NiFe/NF.

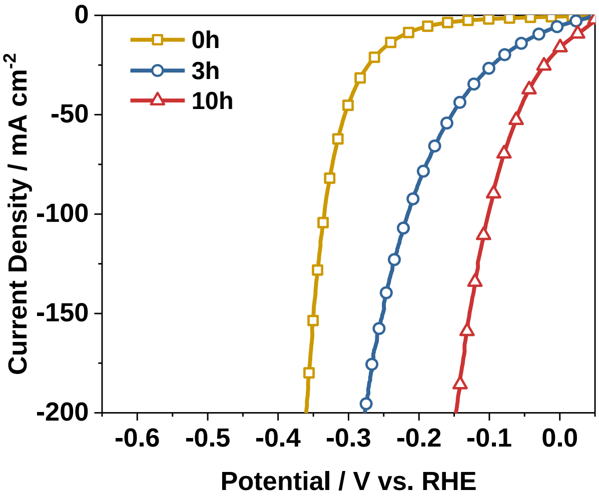

Figure S29 *iR*-Corrected LSV curves of Ni/NiFeMoO$_x$/NF transformed from NiFeMo/NF-Pre obtained at various reaction duration (Figure S4).

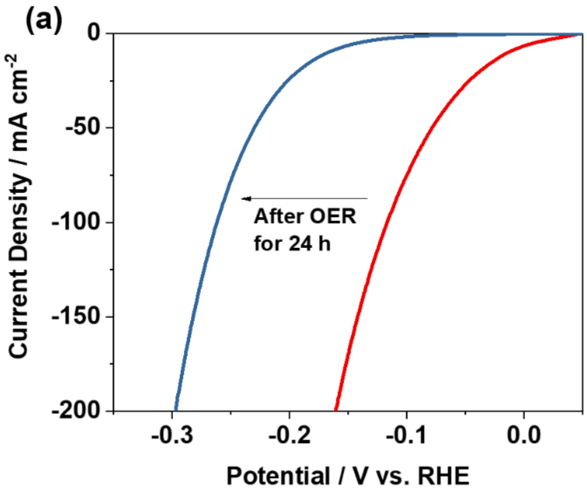

Figure S30 *iR*-Corrected LSV curves of Ni/NiFeMoO$_x$/NF for HER before and after oxygen evolution (Figure 4b in the main text).

After continuous oxygen evolution for 24 h, the Mo content of the electrode was reduced from 73% to 1.7%, and the Mo^{5+} and O vacancies are removed at the same time (Figure S42). Correspondingly, the HER activity was obviously degraded, indicating the important role of Mo^{5+} and O vacancies species in the HER process.

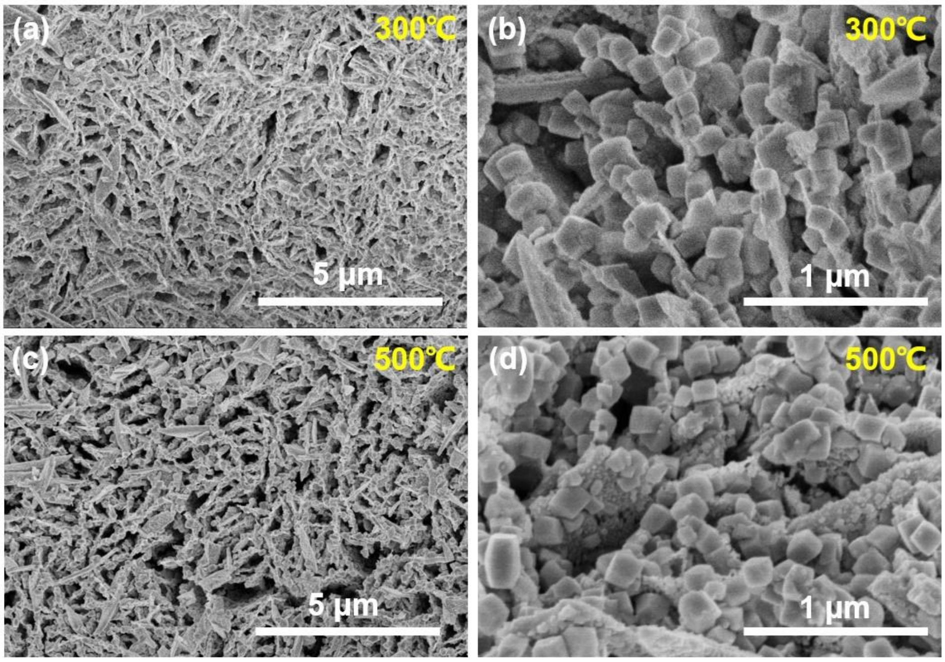

Figure S31 SEM images of Ni-Fe-Mo electrode reduced at (a, b) 300°C and (c, d) 500°C.

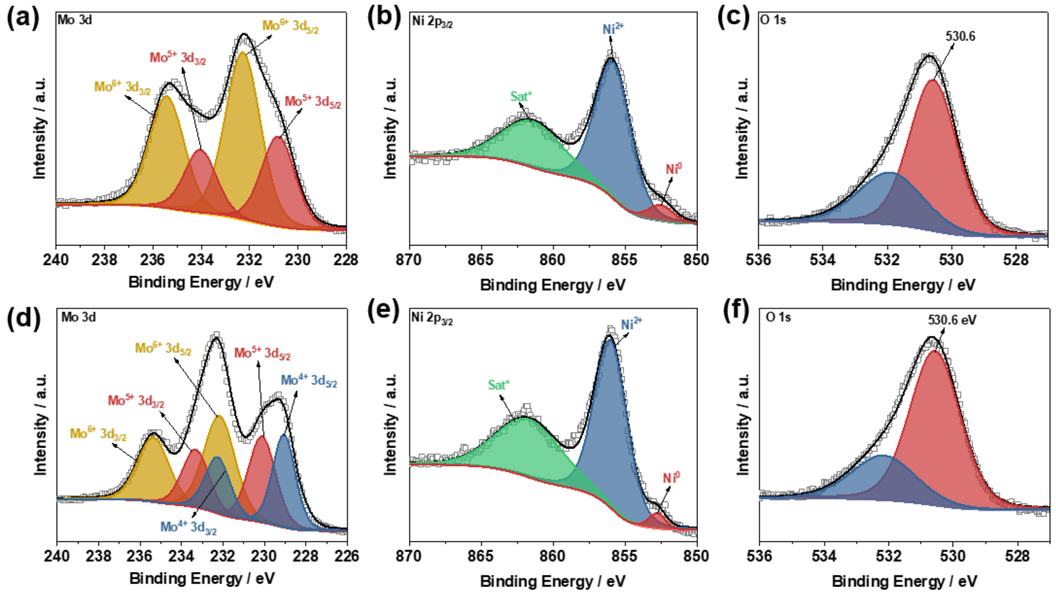

Figure S32 XPS spectra of Ni-Fe-Mo electrode reduced at (a-c) 300°C and (d-f) 500°C.

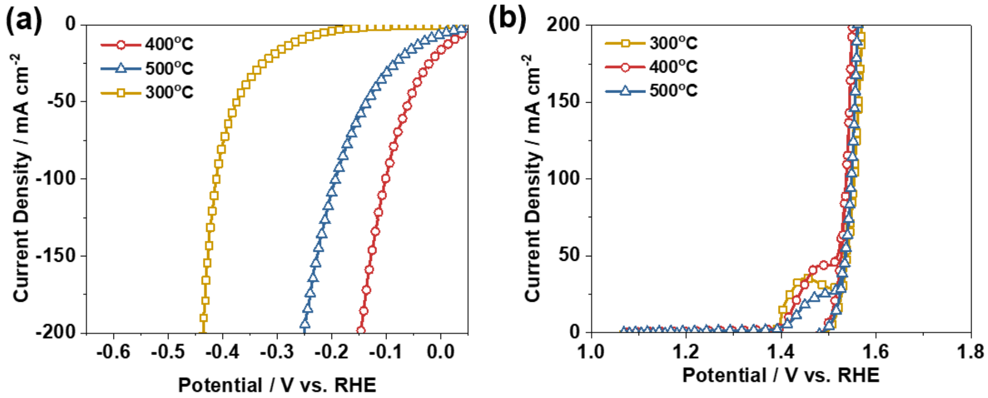

Figure S33 *iR*-Corrected (a) LSV curves for HER and (b) CV curves for OER of Ni-Fe-Mo electrode reduced at different temperature.

The ratio of Mo^{5+} in Mo element of Ni-Fe-Mo electrode obtained at 300°C is 31.5%, which is much smaller than that of electrode obtained at 400°C (59.2%, Figure 2b in the main text). The electrode prepared at 500°C was over reduced, because Mo^{4+} was observed, also giving rise to a lower content of Mo^{5+} species (31.1%). As a result, both of the electrodes obtained at 300 and 500°C showed poorer activity towards HER in comparison with that of 400°C.

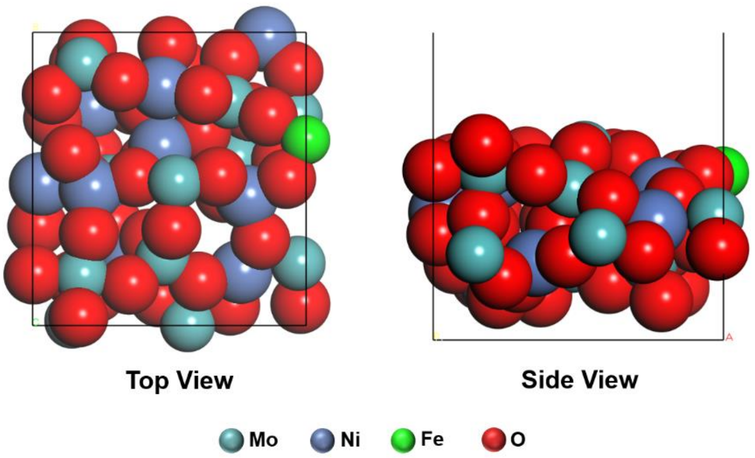

Figure S34 Top and side view of Mo-Ni-Fe-O model that simulates $NiFeMoO_x$.

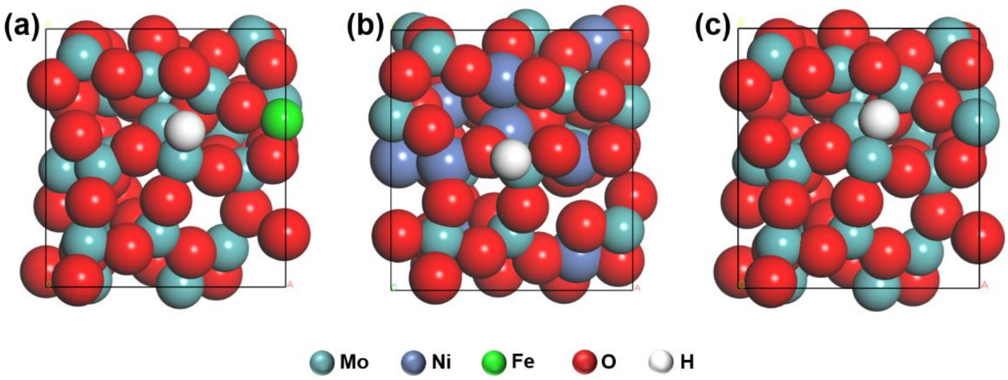

Figure S35 The calculated configuration of H* adsorbed on Mo in (a) Mo-Fe-O, (b) Mo-Ni-O and (c) Mo-O system.

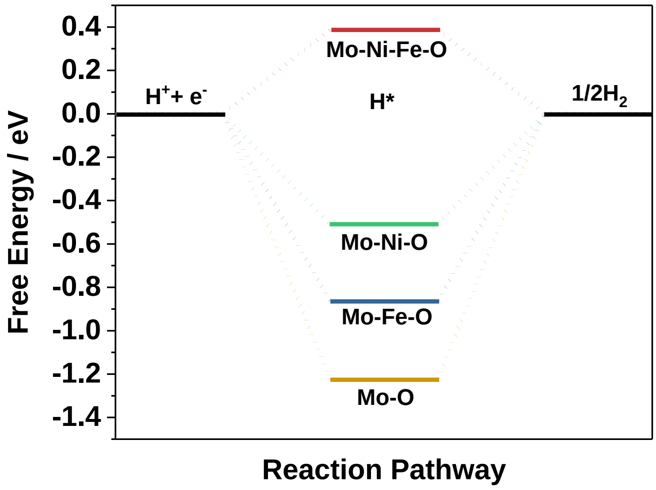

Figure S36 The calculated adsorption free energy of H* on Mo in Mo-Ni-Fe-O, Mo-Fe-O, Mo-Ni-O and Mo-O system.

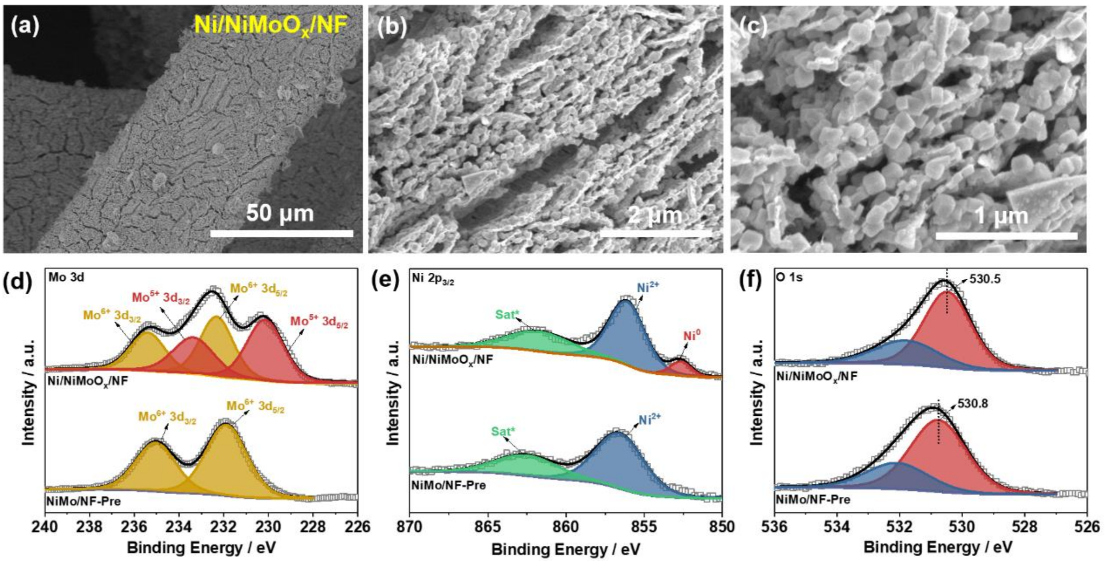

Figure S37 (a-c) SEM images and (d-f) XPS spectra of Ni/NiMoO$_x$/NF.

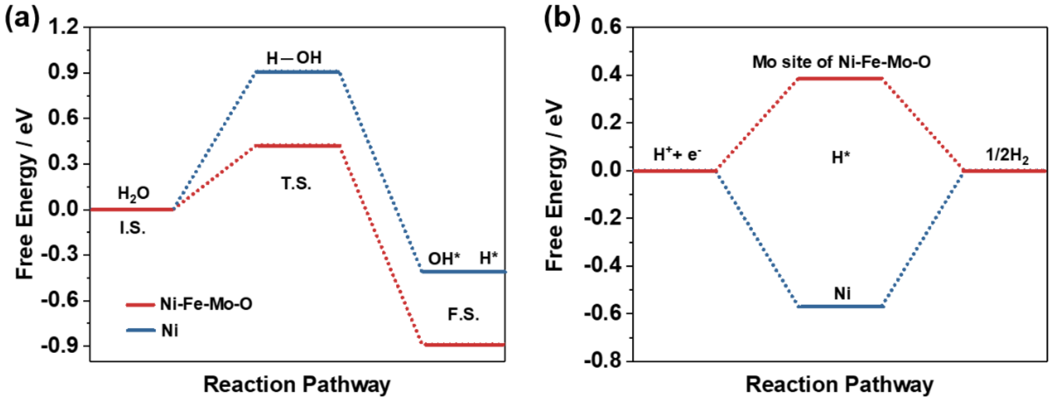

Figure S38 The free energy diagram for (a) H_2O dissociation and (b) H_2 formation on Ni-Fe-Mo-O system and metallic Ni. The data for metallic Ni come from published literature (Nat. Commun., 2017, 8, 15437).

Figure S38a shows that the energy barrier for H_2O dissociation on NiFeMoO$_x$ and Ni is 0.42 and 0.91 eV, respectively, indicating that NiFeMoO$_x$ presents faster H_2O dissociation kinetics. Moreover, the hydrogen adsorption free energy on NiFeMoO$_x$ and Ni is 0.39 and -0.57 eV, respectively (Figure S38b), which suggests that Ni has no advantage with respect to NiFeMoO$_x$ in terms of H_2 formation. Based on the above results, both of the two steps of HER are more facile on NiFeMoO$_x$, and thus the synergistic effect between NiFeMoO$_x$ and

Ni should not be significant. As a result, the main reason for the enhanced HER kinetics on Ni/NiFeMoO$_x$/NF is not resulted from synergistic effect.

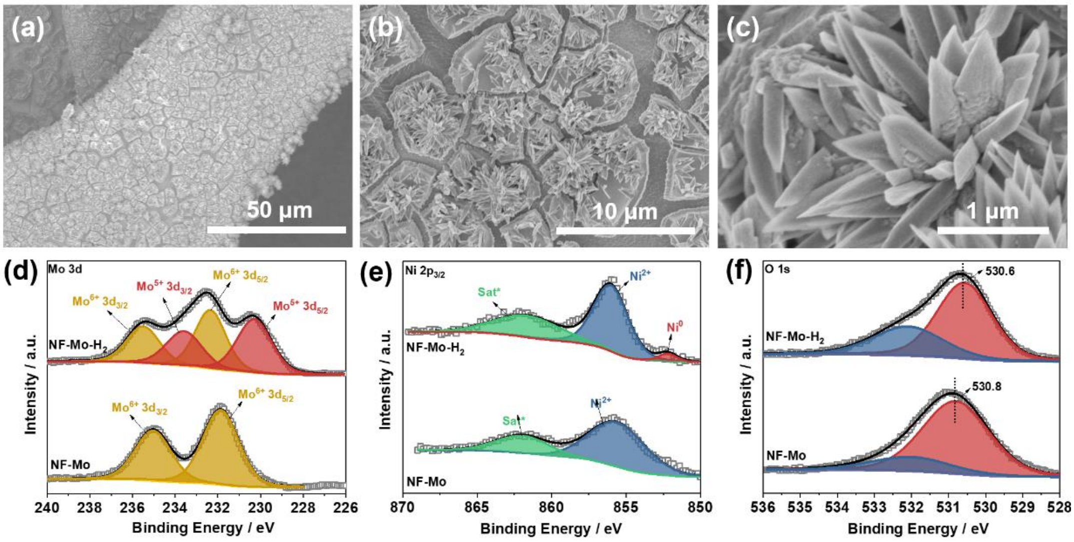

Figure S39 (a-c) SEM images and (d-f) XPS spectra of NF-Mo-H_2 electrode.

It was found that the deposit of NF-Mo electrode was shrunk to pieces that locate independently on the NF substrate after H_2 reduction. The XRD pattern of NF-Mo-H_2 shows a distinct peak at ~44°, ascribed to the (111) plane of FCC Ni (PDF#04-0850) (Figure S18). The XPS shows the presence of nonstoichiometric MoO$_x$ and Ni(0) species in NF-Mo-H_2 electrode.

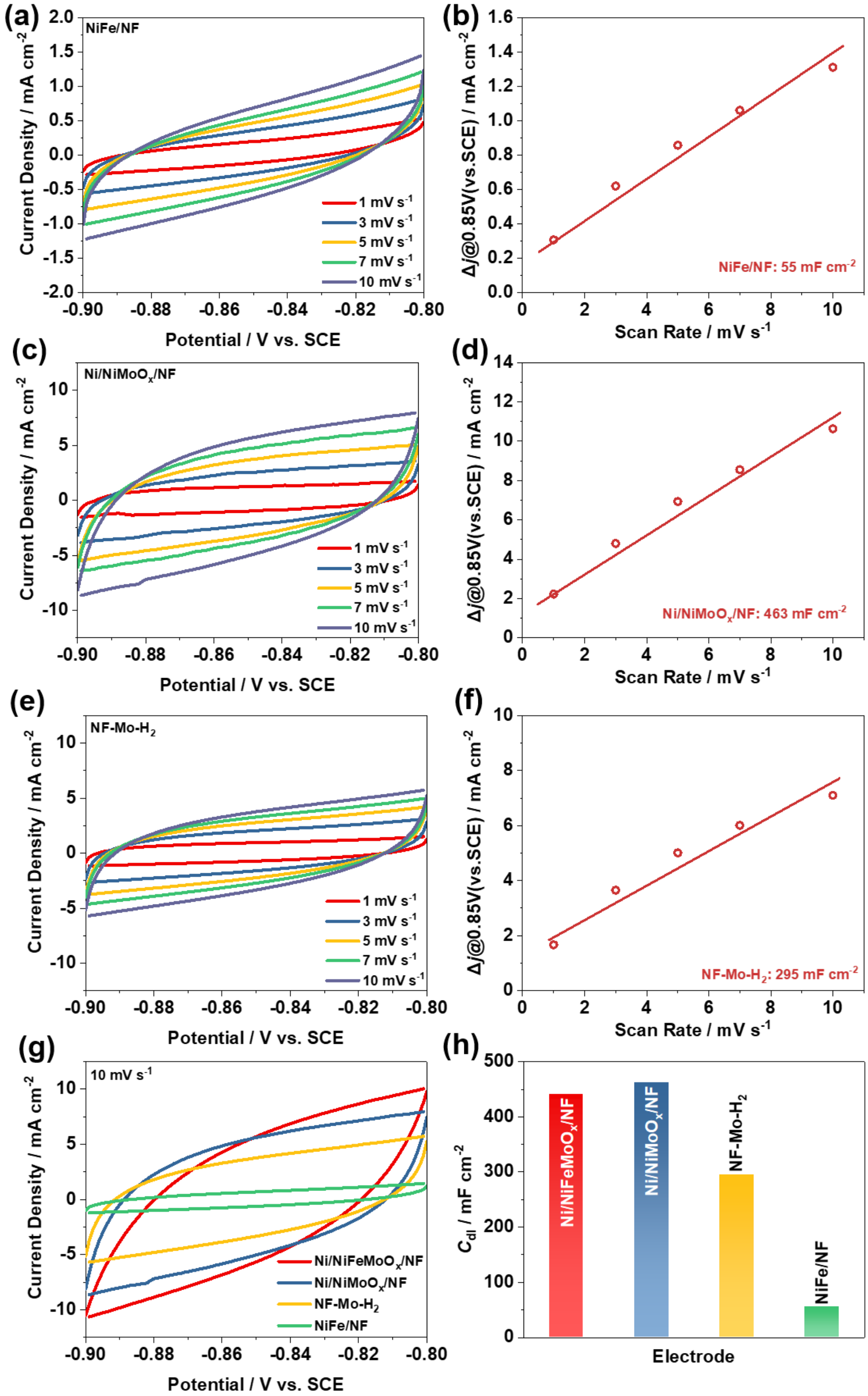

Figure S40 CV curves and C_{dl} of (a, b) NiFe/NF, (c, d) Ni/NiMoO$_x$/NF and (e, f) NF-Mo-H$_2$ electrode. Comparison of (g) CV curves and (h) C_{dl} for NiFe/NF, Ni/NiMoO$_x$/NF, Ni/NiFeMoO$_x$/NF and NF-Mo-H$_2$ electrode. The value of C_{dl} is half of the plot slope.

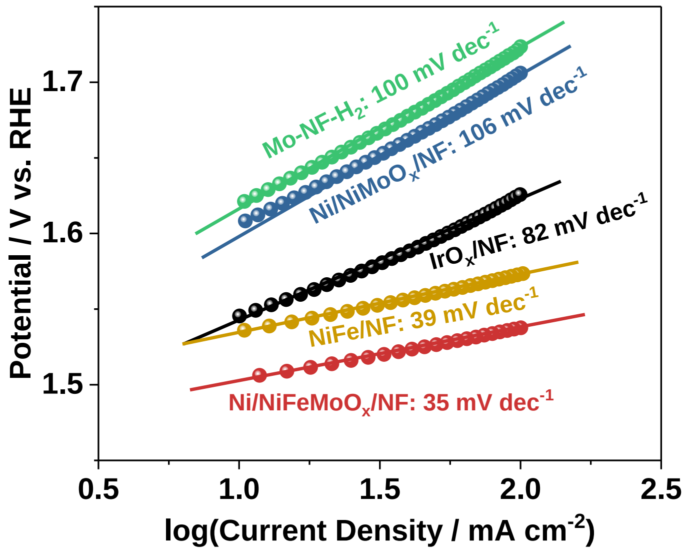

Figure S41 Tafel plots for various electrodes during OER process.

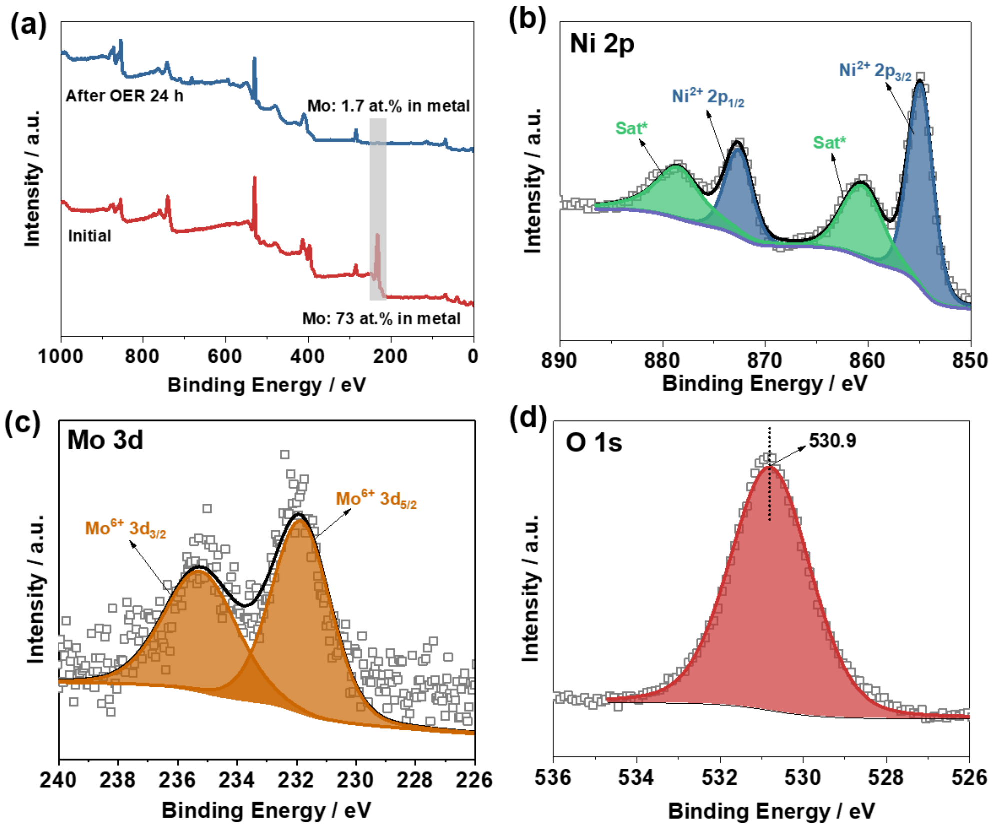

Figure S42 XPS spectra of Ni/NiFeMoO$_x$/NF electrode after oxygen evolution for 24 h.

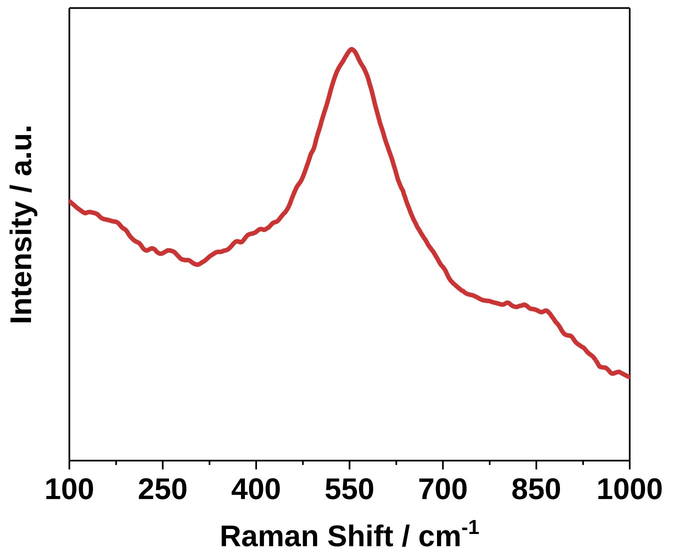

Figure S43 Raman spectrum of Ni/NiFeMoO$_x$/NF electrode after oxygen evolution for 24 h.

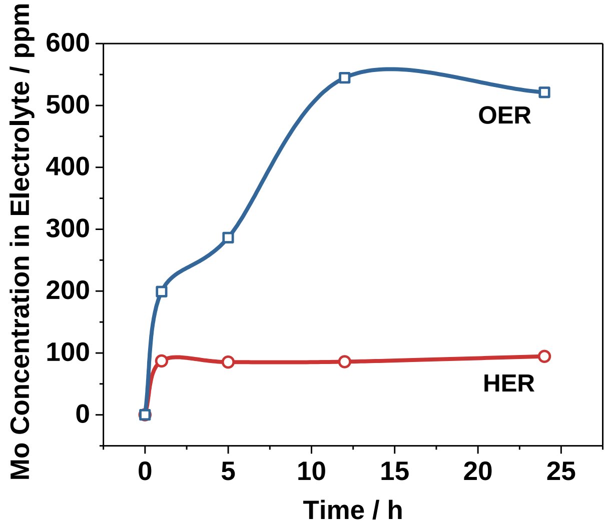

Figure S44 Mo concentration in the electrolyte measured by ICP-MS during continuous HER and OER process.

Mo species has been proved to be crucial for HER, but it is known that some Mo^{6+} compounds (e.g., MoO_3) can dissolve into alkaline liquor, so it is necessary to test the dissolution rate of Mo in the electrode. We found that the concentration of Mo in the electrolyte was 87 ppm after H_2 evolution for 1 h, but it nearly did not increase from 1 h up to

24 h. The dissolved Mo may come from the unstable components of electrode in the beginning of electrolysis, and the other active Mo species are stable enough, which can be explained by the fact that the low electrode potential during HER keeps Mo species at low valence, thus the formation of Mo^{6+} is avoided and the dissolution of Mo species is then inhibited.

In the process of oxygen evolution, the high electrode potential will oxidize low-valent Mo atoms to Mo^{6+} ions. Subsequently, the resulting Mo^{6+} species will then dissolve in the alkaline electrolyte. The elemental analysis to the electrolyte confirms the obvious dissolution of Mo species during OER process.

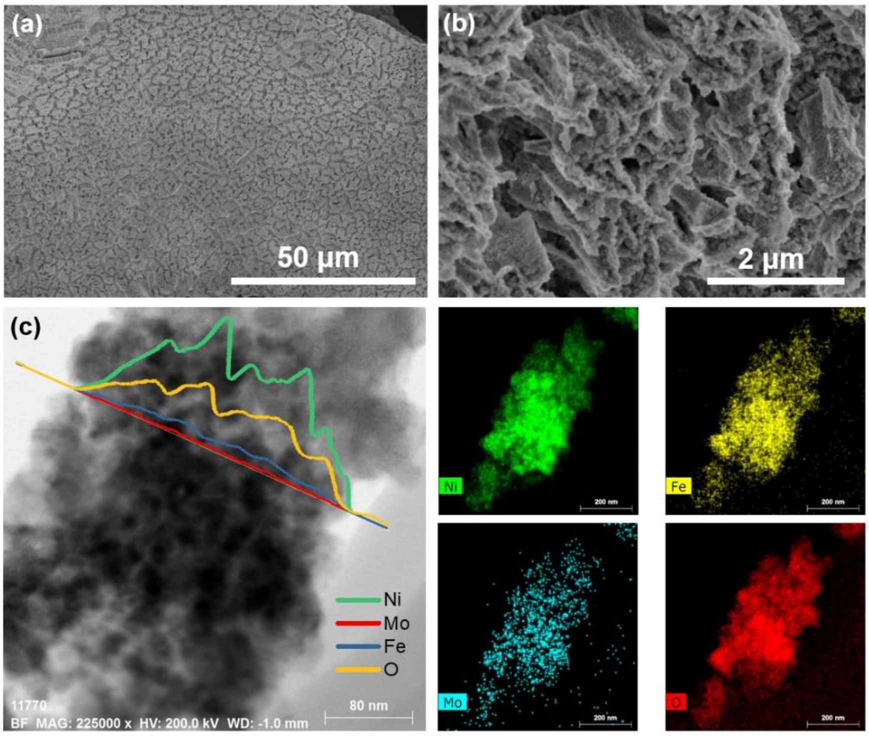

Figure S45 (a, b) SEM images, (c) STEM images and elemental mapping of Ni/NiFeMoO$_x$/NF electrode after oxygen evolution for 24 h.

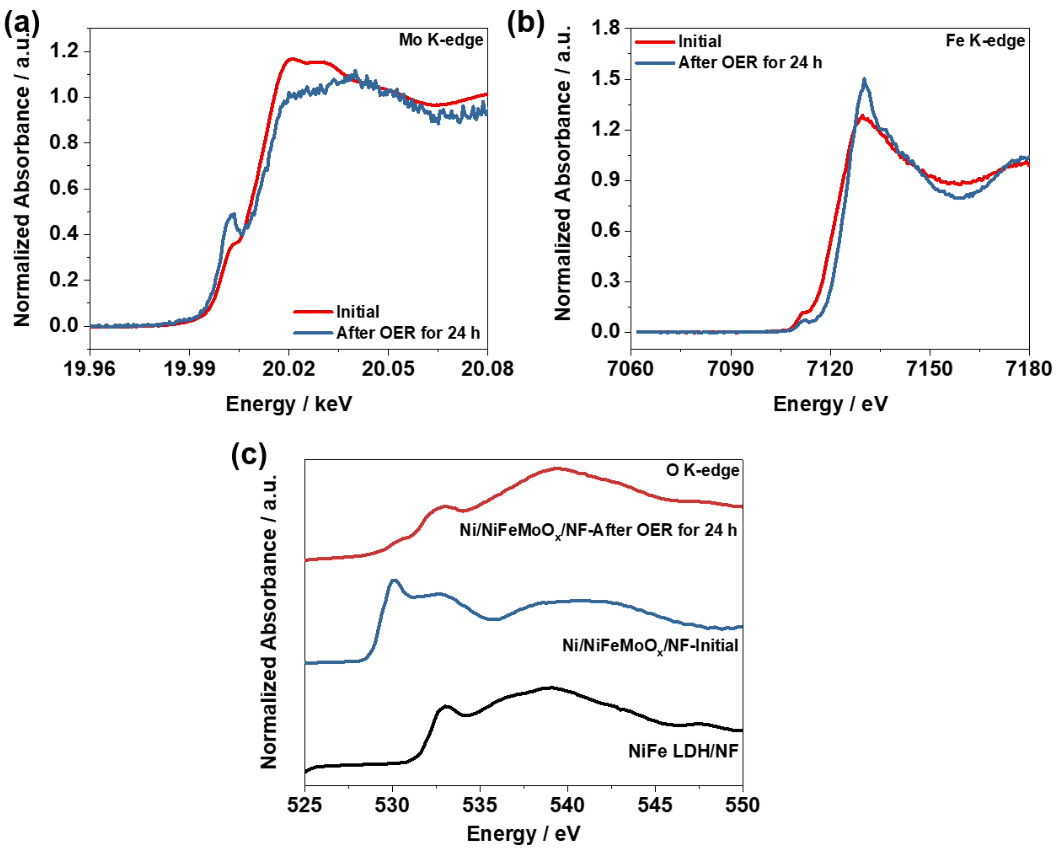

Figure S46 XANES spectra at (a) Mo, (b) Fe and (c) O K-edge for Ni/NiFeMoO$_x$/NF after oxygen evolution for 24 h.

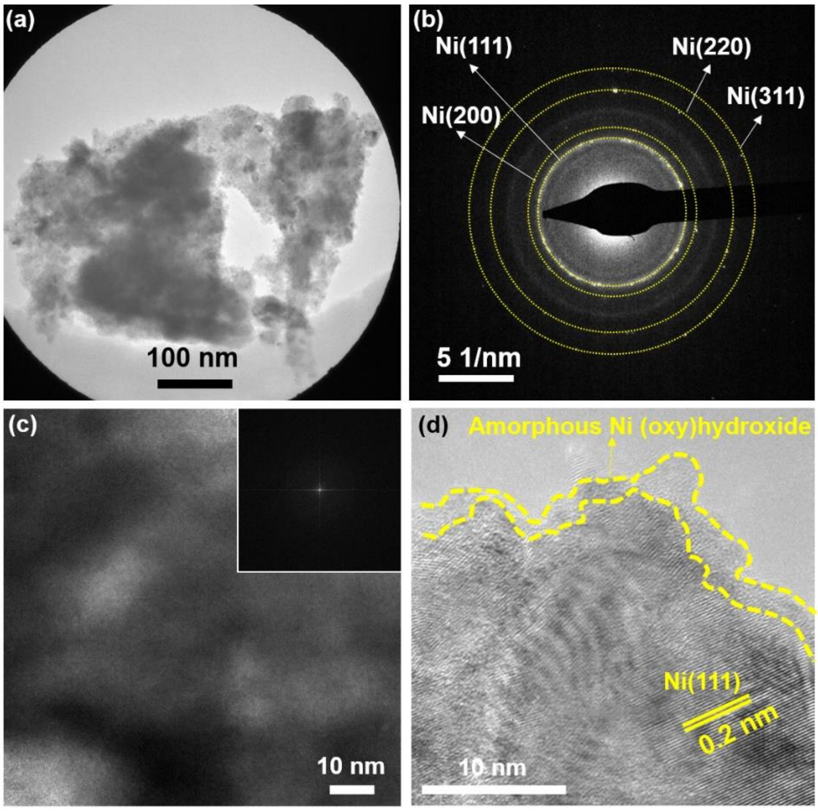

Figure S47 (a) TEM image, (b) SAED, (c-d) HRTEM images of Ni/NiFeMoO$_x$/NF after oxygen evolution for 24 h. The inset in panel (c) shows its corresponding FFT pattern.

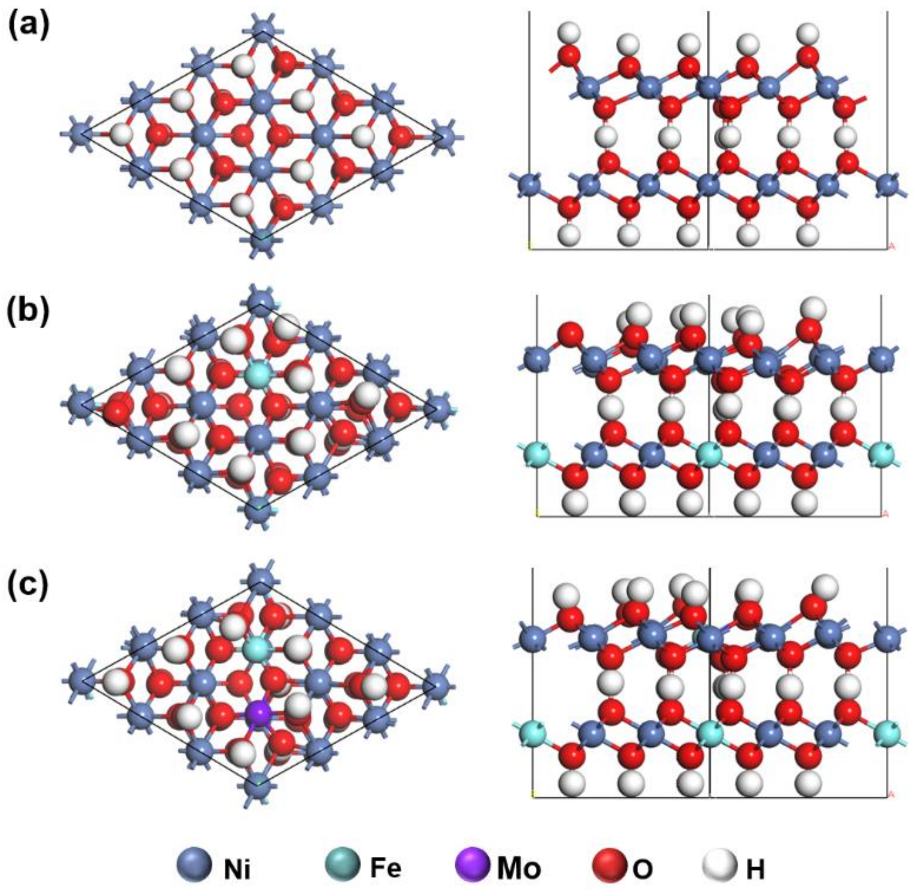

Figure S48 Top and side view of (a) NiOOH, (b) Fe-doped NiOOH and (c) Fe, Mo-codoped NiOOH.

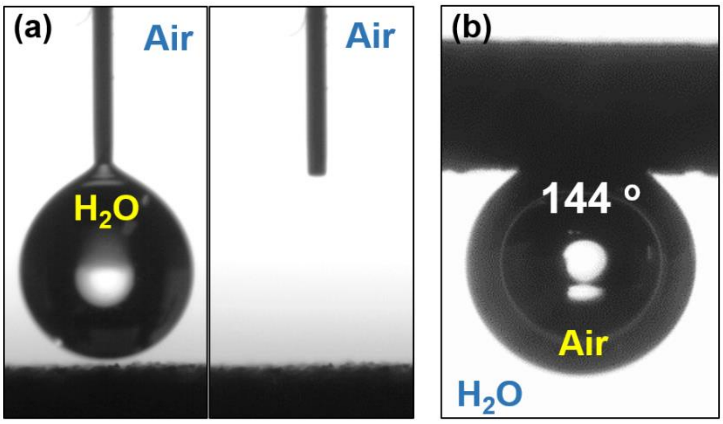

Figure S49 (a) Water contact angle and (b) air bubble contact angle on the surface of Ni/NiFeMoO$_x$/NF.

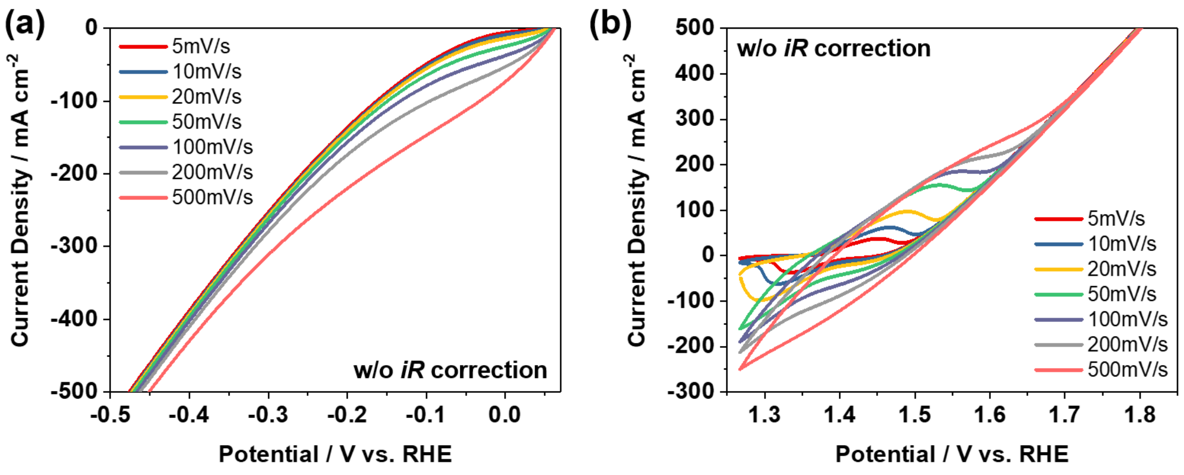

Figure S50 (a) LSV curves for HER and (b) CV curves for OER at different scan rates in 1 M KOH.

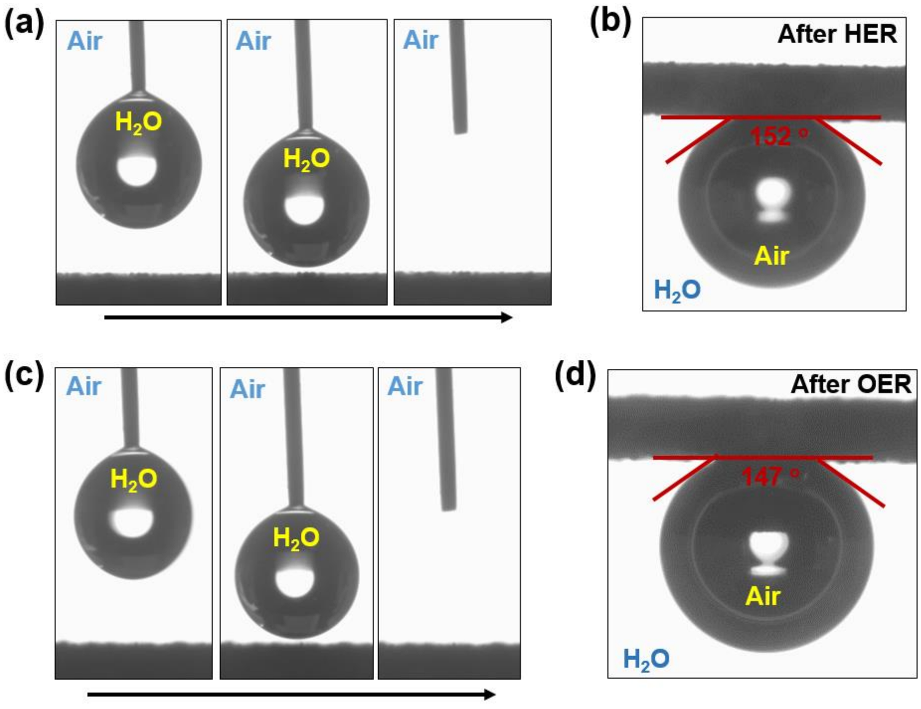

Figure S51 (a) Water contact angle and (b) air bubble contact angle on the surface of Ni/NiFeMoO$_x$/NF after hydrogen evolution for 24 h. (c) Water contact angle and (d) air bubble contact angle on the surface of Ni/NiFeMoO$_x$/NF after oxygen evolution for 24 h.

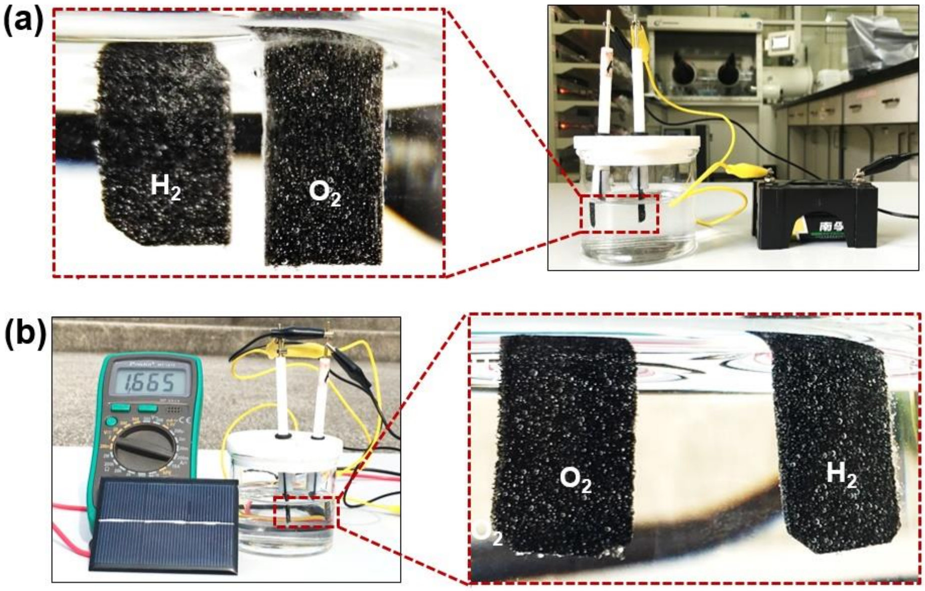

Figure S52 Ni/NiFeMoO$_x$/NF electrolyzer powered by a (a) commercial D-size battery (~1.5 V) and (b) solar cell in 1 M KOH.

References of Supporting Information

[1] X. Liu, X. Wang, X. Yuan, W. Dong, F. Huang, *J. Mater. Chem. A* **2016**, *4*, 167.
[2] a) Y. Y. Chen, Y. Zhang, X. Zhang, T. Tang, H. Luo, S. Niu, Z. H. Dai, L. J. Wan, J. S. Hu, *Adv. Mater.* **2017**, *29*, 1703311; b) G. Mestl, T. Ilkenhans, D. Spielbauer, M. Dieterle, O. Timpe, J. Kröhnert, F. Jentoft, H. Knözinger, R. Schlögl, *Appl. Catal., A* **2001**, *210*, 13; c) S. Boujday, J. Blanchard, R. Villanneau, J. M. Krafft, C. Geantet, C. Louis, M. Breysse, A. Proust, *ChemPhysChem* **2007**, *8*, 2636.
[3] T. Ressler, O. Timpe, T. Neisius, J. Find, G. Mestl, M. Dieterle, R. Schlögl, *J. Catal.* **2000**, *191*, 75.
[4] S. Chaturvedi, J. A. Rodriguez, J. L. Brito, *Catal. Lett.* **1998**, *51*, 85.
[5] H. Wang, J. T. Miller, M. Shakouri, C. Xi, T. Wu, H. Zhao, M. C. Akatay, *Catal. Today* **2013**, *207*, 3.
[6] P. A. Bingham, O. M. Hannant, N. Reeves-McLaren, M. C. Stennett, R. J. Hand, *J. Non-Cryst. Solids* **2014**, *387*, 47.
[7] Y. Wu, G.-D. Li, Y. Liu, L. Yang, X. Lian, T. Asefa, X. Zou, *Adv. Funct. Mater.* **2016**, *26*, 4839.
[8] J. Zhang, T. Wang, D. Pohl, B. Rellinghaus, R. Dong, S. Liu, X. Zhuang, X. Feng, *Angew. Chem.* **2016**, *128*, 6814; *Angew. Chem., Int. Ed.* **2016**, *55*, 6702.
[9] Y. Wang, Y. Sun, F. Yan, C. L. Zhu, P. Gao, X. T. Zhang, Y. J. Chen, *J. Mater. Chem. A* **2018**, *6*, 8479.
[10] Y. Yang, K. Zhang, H. Lin, X. Li, H. C. Chan, L. Yang, Q. Gao, *ACS Catal.* **2017**, *7*, 2357.
[11] F. Qin, Z. Zhao, M. K. Alam, Y. Ni, F. Robles-Hernandez, L. Yu, S. Chen, Z. Ren, Z. Wang, J. Bao, *ACS Energy Lett.* **2018**, *3*, 546.
[12] L. Yang, Z. Guo, J. Huang, Y. Xi, R. Gao, G. Su, W. Wang, L. Cao, B. Dong, *Adv. Mater.* **2017**, *29*, 1704574.
[13] Y. Wu, F. Li, W. Chen, Q. Xiang, Y. Ma, H. Zhu, P. Tao, C. Song, W. Shang, T. Deng, J. Wu, *Adv. Mater.* **2018**, *30*, 1803151.
[14] Y. Jin, H. Wang, J. Li, X. Yue, Y. Han, P. K. Shen, Y. Cui, *Adv. Mater.* **2016**, *28*, 3785.
[15] Y. S. Jin, P. K. Shen, *J. Mater. Chem. A* **2015**, *3*, 20080.
[16] Y. Jin, X. Yue, C. Shu, S. Huang, P. K. Shen, *J. Mater. Chem. A* **2017**, *5*, 2508.
[17] Q. Zhang, P. Li, D. Zhou, Z. Chang, Y. Kuang, X. Sun, *Small* **2017**, *13*, 1701648.
[18] J. Hou, Y. Wu, S. Cao, Y. Sun, L. Sun, *Small* **2017**, *13*, 1702018.
[19] L. Yu, I. K. Mishra, Y. Xie, H. Zhou, J. Sun, J. Zhou, Y. Ni, D. Luo, F. Yu, Y. Yu, S. Chen, Z. Ren, *Nano Energy* **2018**, *53*, 492.
[20] Y. Liu, Q. Li, R. Si, G. D. Li, W. Li, D. P. Liu, D. Wang, L. Sun, Y. Zhang, X. Zou, *Adv. Mater.* **2017**, *29*, 1606200.
[21] C. Du, L. Yang, F. L. Yang, G. Z. Cheng, W. Luo, *ACS Catal.* **2017**, *7*, 4131.
[22] X. Zhang, F. Zhou, W. Pan, Y. Liang, R. Wang, *Adv. Funct. Mater.* **2018**, *28*, 1804600.
[23] L. Yu, H. Zhou, J. Sun, F. Qin, F. Yu, J. Bao, Y. Yu, S. Chen, Z. Ren, *Energy Environ. Sci.* **2017**, *10*, 1820.
[24] D. Senthil Raja, X.-F. Chuah, S.-Y. Lu, *Adv. Energy Mater.* **2018**, *8*, 1801065.
[25] G. Chen, T. Wang, J. Zhang, P. Liu, H. Sun, X. Zhuang, M. Chen, X. Feng, *Adv. Mater.* **2018**, *30*, 1706279.
[26] T. Tang, W.-J. Jiang, S. Niu, N. Liu, H. Luo, Y.-Y. Chen, S.-F. Jin, F. Gao, L.-J. Wan, J.-S. Hu, *J. Am. Chem. Soc.* **2017**, *139*, 8320.
[27] X. Peng, A. M. Qasim, W. Jin, L. Wang, L. Hu, Y. Miao, W. Li, Y. Li, Z. Liu, K. Huo, K.-y. Wong, P. K. Chu, *Nano Energy* **2018**, *53*, 66.

[28] H. Yan, Y. Xie, A. Wu, Z. Cai, L. Wang, C. Tian, X. Zhang, H. Fu, *Adv. Mater.* **2019**, *31*, 1901174.
[29] X. X. Zou, Y. Y. Wu, Y. P. Liu, D. P. Liu, W. Li, L. Gu, H. Liu, P. W. Wang, L. Sun, Y. Zhang, *Chem* **2018**, *4*, 1139.
[30] H. Sun, Y. B. Lian, C. Yang, L. K. Xiong, P. W. Qi, Q. Q. Mu, X. H. Zhao, J. Guo, Z. Deng, Y. Peng, *Energy Environ. Sci.* **2018**, *11*, 2363.
[31] X. R. Zheng, Y. Q. Zhang, H. Liu, D. J. Fu, J. J. Chen, J. H. Wang, C. Zhong, Y. D. Deng, X. P. Han, W. B. Hu, *Small* **2018**, *14*, 1803666.
[32] J. Wang, H.-x. Zhong, Z.-l. Wang, F.-l. Meng, X.-b. Zhang, *ACS Nano* **2016**, *10*, 2342.
[33] N. Han, F. P. Zhao, Y. G. Li, *J. Mater. Chem. A* **2015**, *3*, 16348.
[34] C. Ray, S. C. Lee, K. V. Sankar, B. J. Jin, J. Lee, J. H. Park, S. C. Jun, *ACS Appl. Mater. Interfaces* **2017**, *9*, 37739.
[35] Y. Teng, X.-D. Wang, J.-F. Liao, W.-G. Li, H.-Y. Chen, Y.-J. Dong, D.-B. Kuang, *Adv. Funct. Mater.* **2018**, *28*, 1802463.
[36] Y. Li, L. Hu, W. Zheng, X. Peng, M. Liu, P. K. Chu, L. Y. Suk Lee, *Nano Energy* **2018**, *52*, 360.
[37] J. Zhao, X. Ren, Q. Han, D. Fan, X. Sun, X. Kuang, Q. Wei, D. Wu, *Chem. Commun.* **2018**, *54*, 4987.
[38] Q. Fu, T. Wu, G. Fu, T. Gao, J. Han, T. Yao, Y. Zhang, W. Zhong, X. Wang, B. Song, *ACS Energy Lett.* **2018**, *3*, 1744.
[39] J. Hou, Y. Sun, Y. Wu, S. Cao, L. Sun, *Adv. Funct. Mater.* **2017**, *28*, 1704447.
[40] F. Ming, H. Liang, H. Shi, X. Xu, G. Mei, Z. Wang, *J. Mater. Chem. A* **2016**, *4*, 15148.
[41] F. Yu, H. Zhou, Y. Huang, J. Sun, F. Qin, J. Bao, W. A. Goddard, S. Chen, Z. Ren, *Nat. Commun.* **2018**, *9*, 2551.
[42] H. Wang, H.-W. Lee, Y. Deng, Z. Lu, P.-C. Hsu, Y. Liu, D. Lin, Y. Cui, *Nat. Commun.* **2015**, *6*, 7261.
[43] G. Zhang, Y.-S. Feng, W.-T. Lu, D. He, C.-Y. Wang, Y.-K. Li, X.-Y. Wang, F.-F. Cao, *ACS Catal.* **2018**, *8*, 5431.
[44] F. Yan, Y. Wang, K. Li, C. Zhu, P. Gao, C. Li, X. Zhang, Y. Chen, *Chem.-Eur. J.* **2017**, *23*, 10187.
[45] Z. C. Wu, Z. X. Zou, J. S. Huang, F. Gao, *ACS Appl. Mater. Interfaces* **2018**, *10*, 26283.
[46] Y. Li, H. Zhang, M. Jiang, Q. Zhang, P. He, X. Sun, *Adv. Funct. Mater.* **2017**, *27*, 1702513.
[47] X. G. Wang, W. Li, D. H. Xiong, L. F. Liu, *J. Mater. Chem. A* **2016**, *4*, 5639.
[48] G. Chen, Z. Hu, Y. Zhu, B. Gu, Y. Zhong, H.-J. Lin, C.-T. Chen, W. Zhou, Z. Shao, *Adv. Mater.* **2018**, *30*, 1804333.
[49] W. Li, X. F. Gao, X. G. Wang, D. H. Xiong, P. P. Huang, W. G. Song, X. Q. Bao, L. F. Liu, *J. Power Sources* **2016**, *330*, 156.
